# The Discovery, Validation, and Function of Hypoxia-Related Gene Biomarkers for Obstructive Sleep Apnea

**DOI:** 10.3389/fmed.2022.813459

**Published:** 2022-03-17

**Authors:** Xiaofeng Wu, Zhou Pan, Wei Liu, Shiqian Zha, Yan Song, Qingfeng Zhang, Ke Hu

**Affiliations:** Department of Respiratory and Critical Care Medicine, Renmin Hospital of Wuhan University, Wuhan, China

**Keywords:** obstructive sleep apnea, hypoxia, machine learning, diagnosis, AREG, ATF3, ZFP36, DUSP1

## Abstract

While there is emerging evidence that hypoxia critically contributes to the pathobiology of obstructive sleep apnea (OSA), the diagnostic value of measuring hypoxia or its surrogates in OSA remains unclear. Here we investigated the diagnostic value of hypoxia-related genes and explored their potential molecular mechanisms of action in OSA. Expression data from OSA and control subjects were downloaded from the Gene Expression Omnibus database. Differentially-expressed genes (DEGs) between OSA and control subjects were identified using the *limma* R package and their biological functions investigated with the *clusterProfiler* R package. Hypoxia-related DEGs in OSA were obtained by overlapping DEGs with hypoxia-related genes. The diagnostic value of hypoxia-related DEGs in OSA was evaluated by receiver operating curve (ROC) analysis. Random forest (RF) and lasso machine learning algorithms were used to construct diagnostic models to distinguish OSA from control. Geneset enrichment analysis (GSEA) was performed to explore pathways related to key hypoxia-related genes in OSA. Sixty-three genes associated with hypoxia, transcriptional regulation, and inflammation were identified as differentially expressed between OSA and control samples. By intersecting these with known hypoxia-related genes, 17 hypoxia-related DEGs related to OSA were identified. Protein-protein interaction network analysis showed that 16 hypoxia-related genes interacted, and their diagnostic value was further explored. The 16 hypoxia-related genes accurately predicted OSA with AUCs >0.7. A lasso model constructed using *AREG, ATF3, ZFP36*, and *DUSP1* had a better performance and accuracy in classifying OSA and control samples compared with an RF model as assessed by multiple metrics. Moreover, GSEA revealed that *AREG, ATF3, ZFP36*, and *DUSP1* may regulate OSA *via* inflammation and contribute to OSA-related cancer risk. Here we constructed a reliable diagnostic model for OSA based on hypoxia-related genes. Furthermore, these transcriptional changes may contribute to the etiology, pathogenesis, and sequelae of OSA.

## Introduction

Obstructive sleep apnea (OSA), characterized by partial or total collapse of the upper airway during sleep, is a major and underappreciated public health problem. Untreated OSA patients are at significantly increased risk of multiple diseases and traffic accidents, and the economic burden of OSA increases with disease severity (1, 2). The prevalence of OSA is increasing as the global obesity epidemic worsens, and OSA currently affects 9% of women and 24% of men (3). The gold standard clinical diagnosis of OSA is based on polysomnography (PSG), but due to the need for specialist equipment, long-term continuous monitoring, and specialist input and training, PSG is not widely carried out in the community. Although other methods of risk stratification and screening for OSA exist, such as the Berlin questionnaire, expiratory NO detection and face recognition technology, these approaches are not recommended for the diagnosis and treatment of OSA (4). Furthermore, while home sleep apnea testing has high sensitivity [79% (95% CI, 71–86%)] and specificity [79% (95% CI, 63–89%)], it still takes all night and is costly (5).Therefore, new, low-cost, easily applicable diagnostic methods for OSA are required to replace the need for PSG.

Hypoxia is best defined as an imbalance between oxygen supply and demand, which varies widely depending on the organ or tissue in the body (6). The intermittent hypoxia (IH) caused by sleep apnea characterizes OSA (7). Similar to ischemia-reperfusion injury, chronic intermittent hypoxia (CIH) can lead to abundant reactive oxygen species (ROS) formation through repeated cycles of systemic hypoxia/reoxygenation (8) and increase blood pressure by regulating sympathetic activity (9). In both animal and human experiments, nerve damage and the slow autonomic response system caused by IH in OSA result in an ability to restore cardiovascular regulation (10). In addition, OSA-related IH is a risk factor for the development and progression of lung cancer. Thus, investigating the molecular basis of hypoxia and its effects in OSA could improve our understanding of the pathogenesis of OSA and related diseases (11–13).

Therefore, here we evaluated hypoxia-related gene expression in OSA to (i) explore the diagnostic value of hypoxia-related genes in OSA patients; and (ii) investigate their possible contribution to OSA pathobiology using *in silico* analyses.

## Materials and Methods

### Data Sources

The transcriptomic profiles of 34 OSA and eight control subjects were retrieved from GSE135917 in the Gene Expression Omnibus (GEO) database (www.ncbi.nlm.nih.gov/geo/). In this public dataset, the diagnosis of OSA patients were based on AHI≥30 events/h or RDI≥5 events/h, and the control subjects had normal AHI or RDI. Thirty-four OSA subjects, including 16 males and 18 females, had a mean age of 51.3 years with a mean BMI of 40.7 kg/m^2^. When diagnosis, 10 OSA patients were enrolled by RDI and their average RDI was 19.2 events/h, while the other 24 OSA patients were recruited by AHI with the average of 41.5 events/h. Besides, eight OSA subjects had diabetes mellitus, 12 subjects had hypertension disease, and two subjects had heart disease. Control subjects, including one male and seven females, had a mean age of 54.5 years with a mean BMI of 35.2 kg/m^2^. Among them, three control subjects had hypertension disease. Two hundred hypoxia-related genes were obtained from the HALLMARK_HYPOXIA gene set in the MSigDB database (www.gsea-msigdb.org/gsea/msigdb/cards/HALLMARK_HYPOXIA.html). The GSE135917 dataset was used to detect hypoxia-related genes involved in OSA, construct a diagnostic model, and identify potential diagnostic biomarkers for OSA. Ten OSA and eight control subjects from GSE38792 were used to validated the OSA diagnostic model.

### Identification of Hypoxia-Related Genes Involved in OSA

Differentially-expressed genes (DEGs) between OSA and control subjects in GSE135917 were identified using the *limma* package in R with the threshold |log_2_ FC| >1 and *p* < 0.05. The functional enrichment of DEGs in biological pathways and processes {according to the Gene Ontology [GO; biological process (BP), cellular component (CC), and molecular function (MF)] and KEGG databases} was analyzed using *clusterProfiler* in R. Thereafter, hypoxia-related genes involved in OSA were identified by overlapping DEGs with the 200 hypoxia-related genes identified in MSigDB. Interactions between hypoxia-related genes involved in OSA were investigated by inputting them into the STRING database (www.string-db.org/) to construct a protein-protein interaction (PPI) network, and genes within the PPI network were evaluated by correlation analysis.

### Identification of Potential Diagnostic Biomarkers for OSA

The diagnostic value of genes within the PPI network was evaluated by area under the receiver operating characteristic (AUC-ROC) curves. Hypoxia-related genes involved in OSA with AUCs >0.7 were further explored as potential diagnostic biomarkers for OSA. Moreover, micro-(mi)RNA-diagnostic biomarker and transcription factor (TF)-diagnostic biomarker regulatory networks were constructed using the *mirnet* database (https://www.mirnet.ca). Furthermore, geneset enrichment analysis (GSEA) was performed using KEGG genesets as reference to reveal potential mechanisms by which the identified diagnostic biomarkers might regulate OSA. Reference genesets were downloaded from the MSigDB database (http://software.broadinstitute.org/gsea/msigdb/index.jsp).

### Identification of Diagnostic Biomarkers and Development of an OSA Diagnostic Model

Machine learning methods including random forest (RF) (14) and lasso regression (15) were applied to construct a diagnostic model based on the OSA diagnostic biomarkers identified in the GSE135917 dataset using the *randomForest* and *glmnet* R packages, respectively. ROC curves and their derived sensitivities, specificities, and AUCs were constructed to evaluate the accuracy of the established diagnostic models. To compare the performance of the RF and lasso algorithms, accuracy, error, precision, recall, F1-score, and Kolmogorov-Smirnov (K-S) values were calculated using the following formulae: accuracy = (TP + TN)/(TP + FP + TN + FN); error = 1-Accuracy; precision = TP/(TP + FP); recall = TP/(TP + FN); F1-score = 2 × precision × recall/(precision + recall); K-S = max[TP/(TP + FN)-FP/(FP + TN)], where TP is true positive, FN is false negative, FP is false positive, and TN is true negative. The diagnostic model showing the best performance for classifying OSA and control subjects was selected and validated in the GSE38792 dataset.

## Results

### DEGs Associated With Hypoxia and Inflammation in OSA

**Figure 1:**
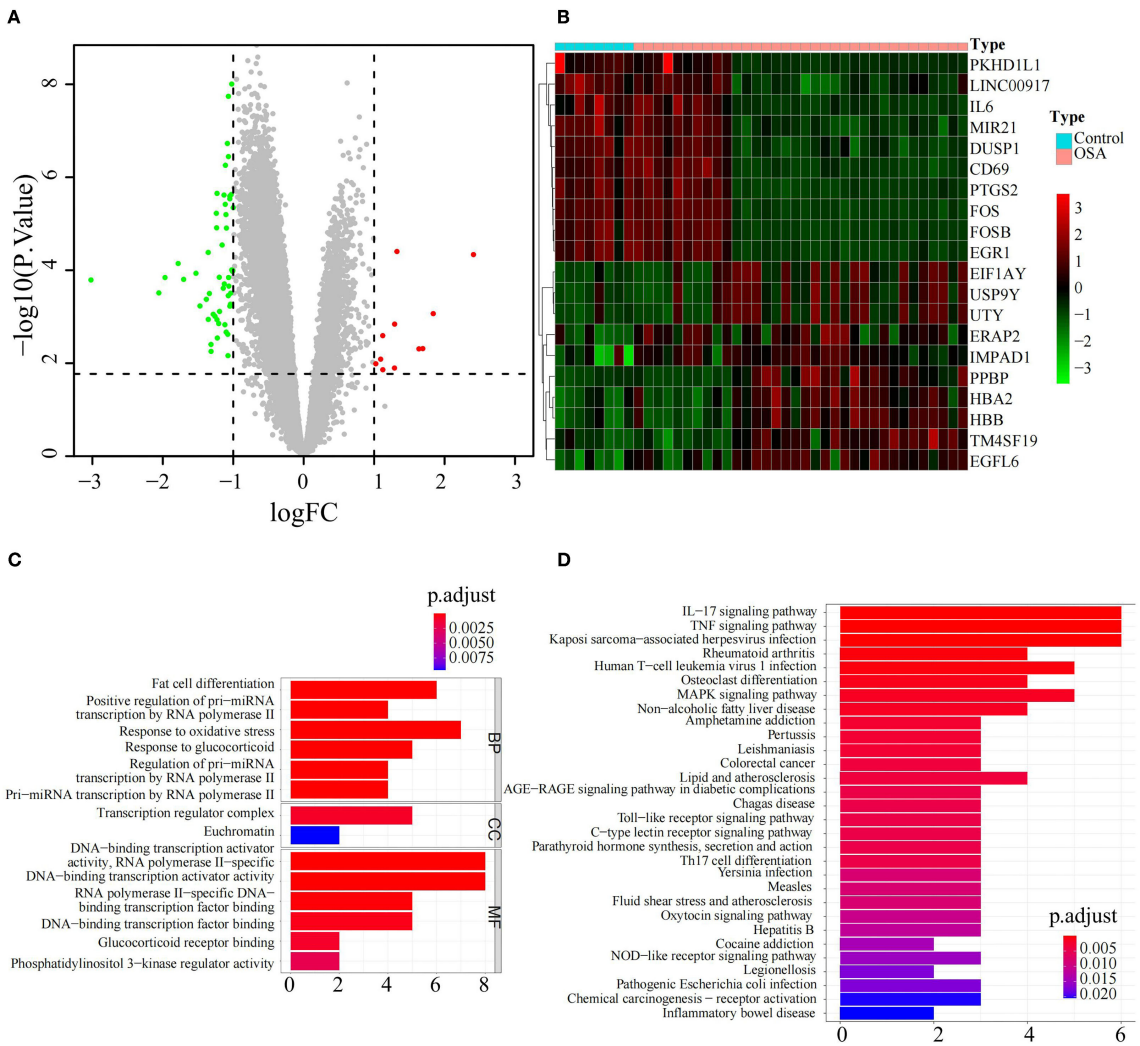
Identification and functional analysis of differentially expressed genes between OSA and control subjects. **(A)** Volcano plot of the DEGs, including 11 upregulated genes in red and 52 downregulated genes in green, between OSA and control subjects. **(B)** Heatmap showing the expression of the top 10 upregulated genes and top 10 downregulated genes in OSA compared with control subjects. **(C)** Bar chart showing the GO enrichment analysis of DEGs, including biological processes, molecular functions, and cellular components. **(D)** Bar chart showing KEGG enrichment analysis of DEGs.

Sixty-three DEGs between OSA and control subjects were identified in GSE135917 (Supplementary Table 1). Among them, 11 genes were upregulated and 52 were downregulated in OSA subjects relative to control (Figure 1A). The expression levels of the top ten upregulated (*EIF1AY, USP9Y, UTY, ERAP2, IMPAD1, PPBP, HBA2, HBB, TM4SF19, EGFL6*) and downregulated (*PKGD1L1, LINC00917, IL6, MIR21, DUSP1, CD69, PTGS2, FOS, FOSB, EGR1*) genes in OSA are shown in the heatmap in Figure 1B. Functional enrichment analysis showed that those DEGs were significantly enriched in 245 BP, 2 CC, 36 MF (Supplementary Table 2), and 53 KEGG pathways (Supplementary Table 3). The top GO terms were associated with hypoxia and transcription such as fat cell differentiation, response to oxidative stress, response to glucocorticoids, transcription regulator complex, DNA-binding transcription activator activity, and DNA-binding transcription factor binding (Figure 1C). Interestingly, DEGs were also involved in inflammation-which has complex interactions with hypoxia-such as IL-17 signaling, TNF signaling, and MAPK signaling (Figure 1D). In addition, there was enrichment for diseases closely associated with OSA such as rheumatoid arthritis and non-alcoholic fatty liver disease (Figure 1D).

### Hypoxia-Related Genes Involved in OSA

**Figure 2:**
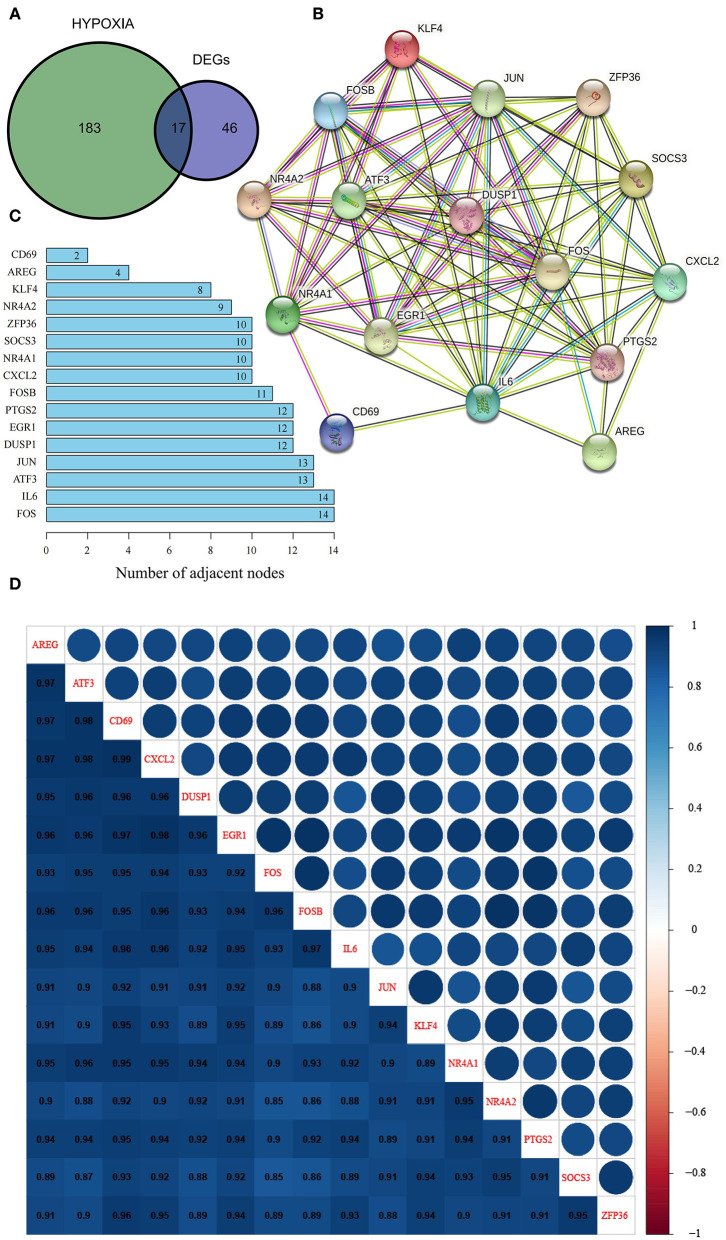
Identification and characterization of hypoxia-related DEGs in OSA. **(A)** Venn diagram of overlapping hypoxia-related genes and DEGs. **(B)** PPI network of hypoxia-related DEGs. **(C)** Number of adjacent nodes of hypoxia-related DEGs in the PPI network. **(D)** Heatmap of correlations between hypoxia-related DEGs in the PPI network.

In view of the identified DEGs and their functional enrichment, we hypothesized that hypoxia plays an important role in OSA. Thus, we extracted 17 hypoxia-related genes involved in OSA by intersecting the 63 DEGs with 200 hypoxia-related genes in MSigDB (Figure 2A); all 17 genes were significantly downregulated in OSA subjects compared with control subjects (Supplementary Figure 1). PPI analysis showed that *FOS, IL6, ATF3, JUN, DUSP1, EGR1, PTGS2, FOSB, CXCL2, NR4A1, SOCS3, ZFP36, NR4A2, KLF4, AREG*, and *CD69* interacted with each other (Figure 2B), with *FOS* and *IL6* probable hub nodes, followed by ATF3 and JUN, since they had the most interactions with hypoxia-related genes involved in OSA (Figure 2C). Moreover, the expression levels of the 16 genes in the PPI network were strongly positively correlated (≥0.85, Figure 2D), further demonstrating their positive interactions.

### Hypoxia-Related Genes are Accurate Biomarkers for the Diagnosis of OSA

**Figure 3:**
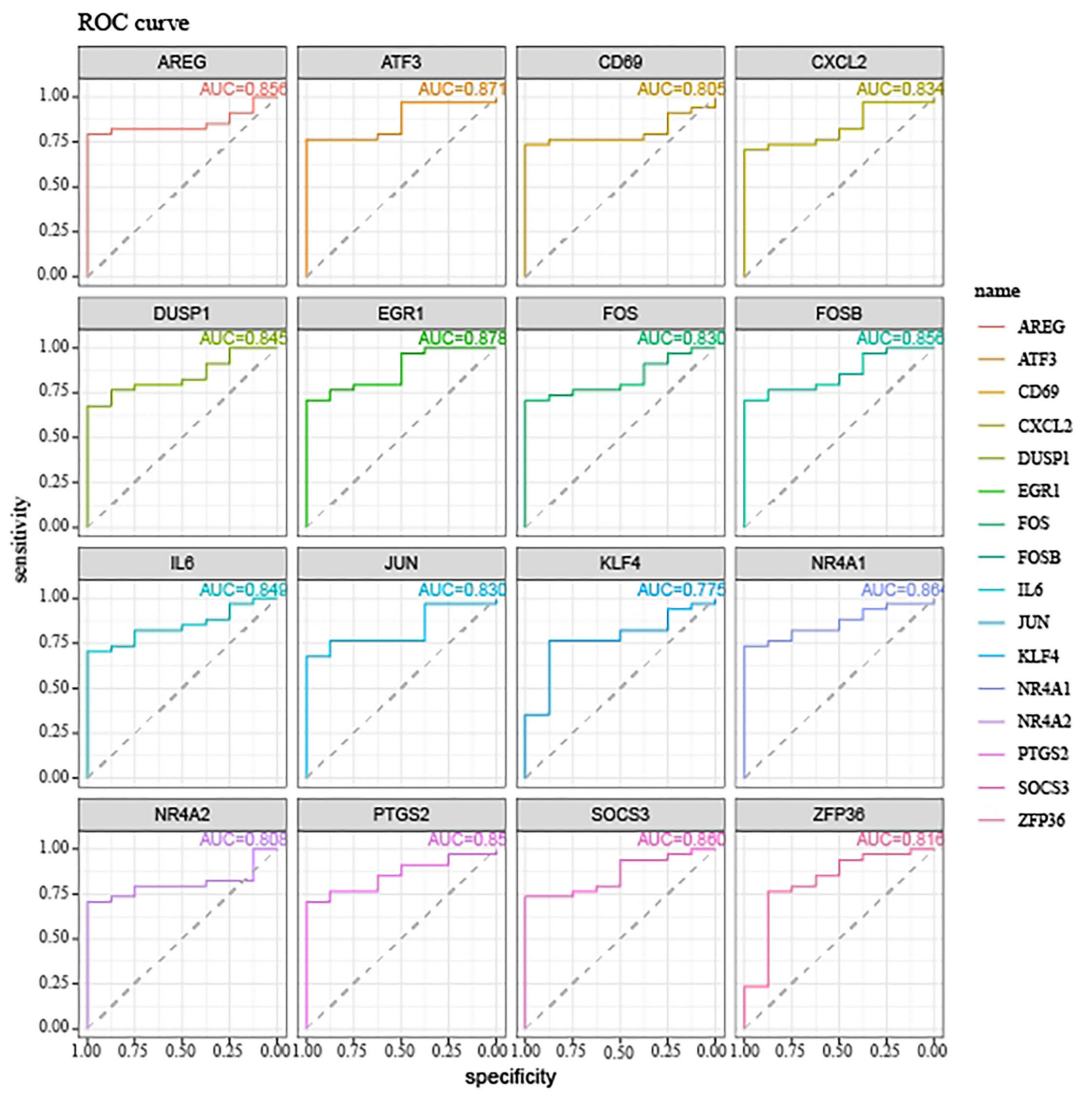
ROC curves showing the diagnostic value of hypoxia-related DEGs in the PPI network.

**Figure 4:**
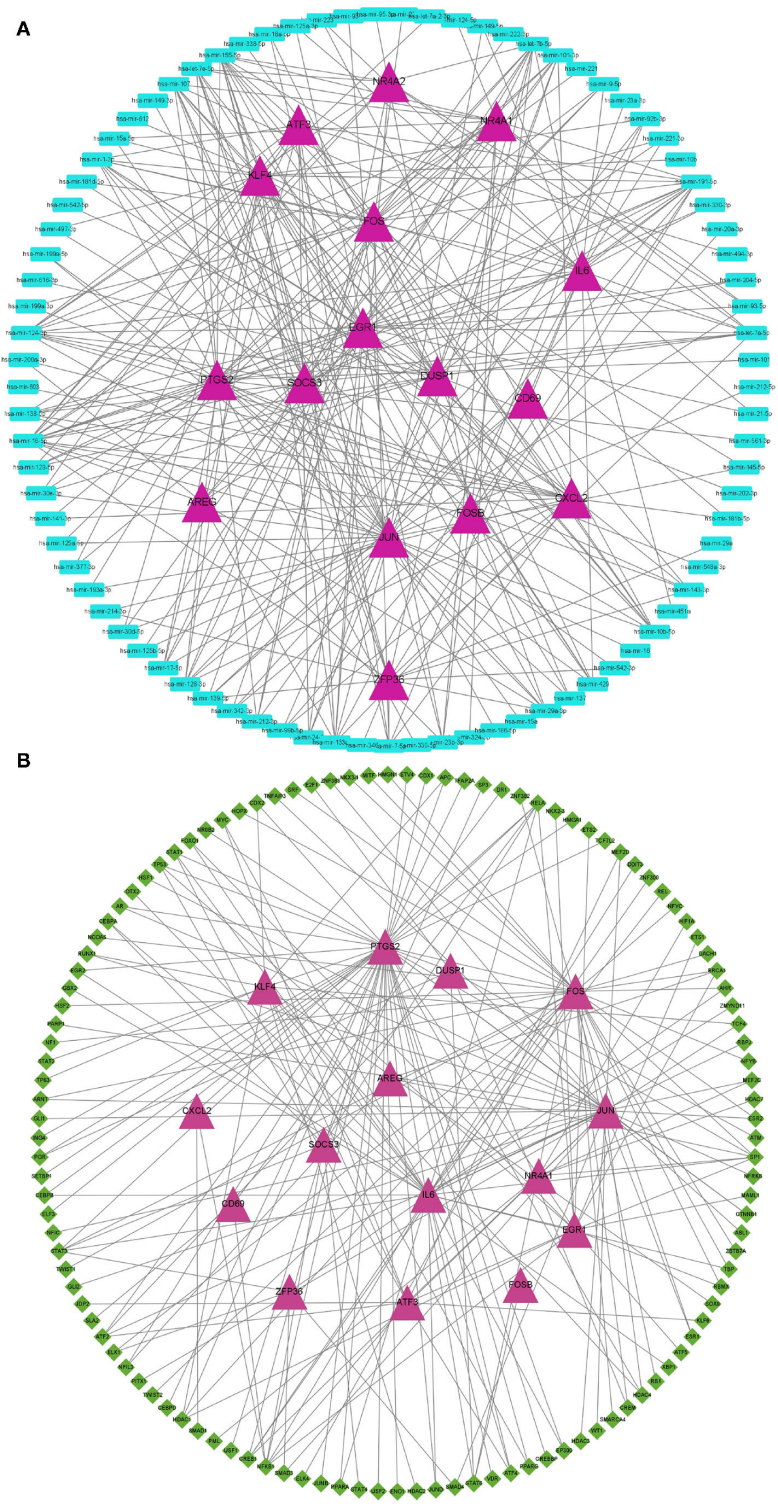
Regulation network of hypoxia-related DEGs in the PPI network. **(A)** Construction of the miRNA-mRNA network. Hypoxia-related DEGs are represented by magenta triangles, and the corresponding miRNAs are represented by blue triangles. **(B)** Construction of the TF-mRNA network. Hypoxia-related DEGs are represented by magenta triangles, and the TFs targeting hypoxia-related DEGs are represented by green diamonds.

We next explored the diagnostic value of the 16 hypoxia-related genes in the PPI network. The AUCs of *FOS, IL6, ATF3, JUN, DUSP1, EGR1, PTGS2, FOSB, CXCL2, NR4A1, SOCS3, ZFP36, NR4A2, KLF4, AREG*, and *CD69* were all >0.7 (Figure 3), indicating that they might be useful diagnostic biomarkers for OSA. Next, we investigated which miRNAs and TFs might regulate their expression. Using *mirnet*, we detected 290 miRNA-diagnostic biomarker pairs, which allowed us to construct an miRNA-mRNA network composed of 16 hypoxia-related genes and 85 miRNAs (Figure 4A). We also identified 198 TF-diagnostic biomarker pairs, which produced a TF-target network composed of 15 diagnostic biomarkers and 118 TFs (Figure 4B).

### Lasso Diagnostic Model for OSA Based on Hypoxia-Related Genes Involved in OSA

**Figure 5:**
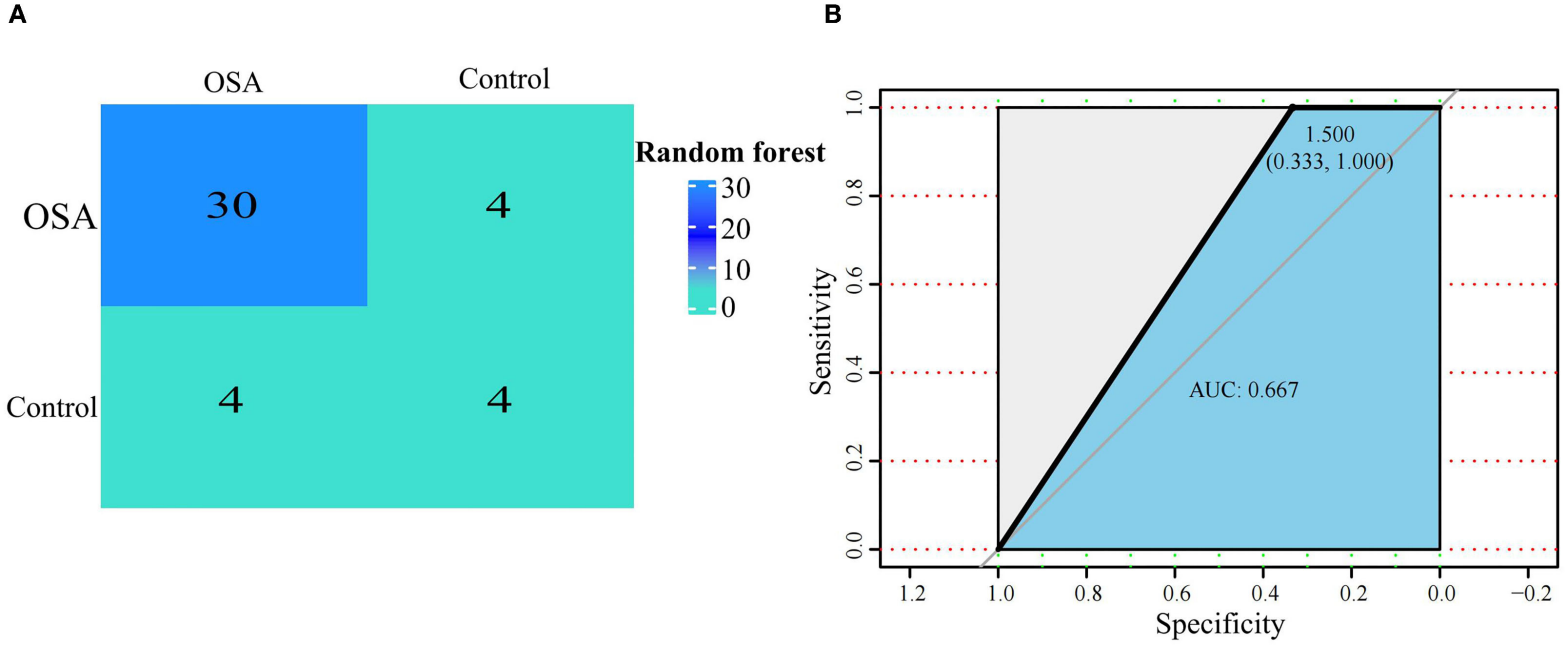
Establishment of a random forest model based on hypoxia-related DEGs in OSA. **(A)** Confusion matrix of GSE135917 using the random forest model. **(B)** The diagnostic performance of the random forest model by ROC curve analysis.

**Figure 6:**
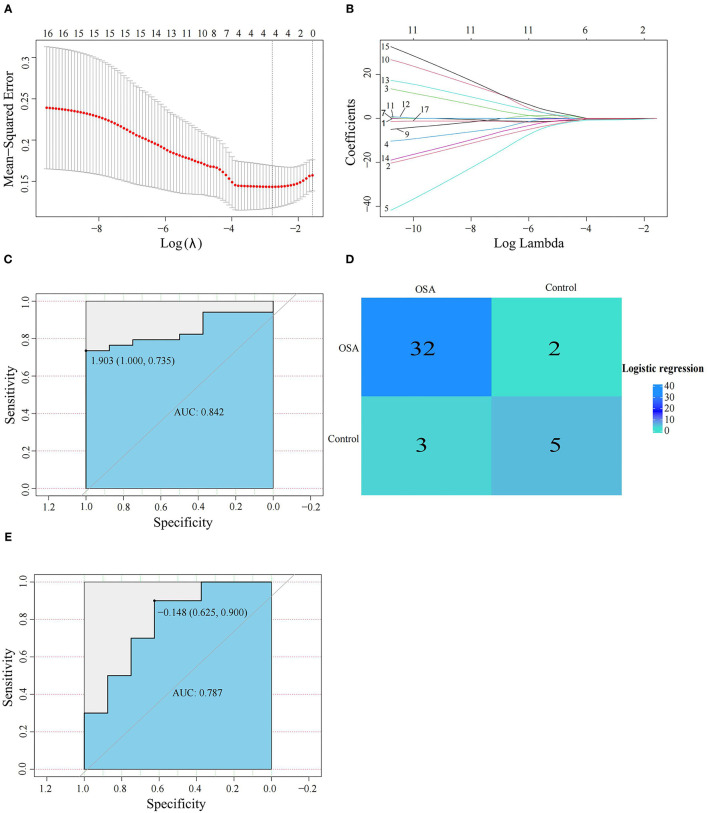
Establishment and verification of the lasso model. **(A,B)** The lasso algorithm was used to screen the gene signature in OSA. **(C)** The diagnostic performance of the lasso model by ROC curve analysis in GSE135917. **(D)** Confusion matrix of GSE135917 using the lasso model. **(E)** Verification of the diagnostic performance of the lasso model by ROC curve analysis in GSE38792.

We next used the 16 hypoxia-related genes in the PPI network to construct OSA diagnostic models. The confusion matrix of the RF model showed correct classification of 30 OSA patients and four control subjects (Figure 5A) with an AUC of 0.667 (Figure 5B). Similarly, using lasso, *AREG, ATF3, ZFP36*, and *DUSP1* were identified as a predictive gene signature (Figures 6A,B), and the diagnostic OSA model based on these four genes had an AUC of 0.842 (Figure 6C). The confusion matrix of the lasso model also demonstrated its superior performance over the RF model, with 32 OSA patients and five control subjects correctly identified (Figure 6D). Moreover, as shown in Table 1, the lasso model had higher precision and K-S values, indicating that it was better able to distinguish OSA from control subjects than the RF model. Therefore, the lasso model was selected as the OSA diagnostic model of choice, with validation in the external dataset showing an AUC of 0.787 (Figure 6E).

**Table 1:** The diagnostic accuracy of the lasso and random forest models.

**Model**	**Precision**	**Recall**	**Fl-score**	**Accuracy**	**Error**	**K-S**
Random forest	0.88	0.88	0.79	0.81	0.19	0.38
Logistic regression	0.91	0.94	0.91	0.88	0.12	0.535

### GSEA Reveals a Potential Role for Hypoxia-Related Genes in OSA

**Figure 7:**
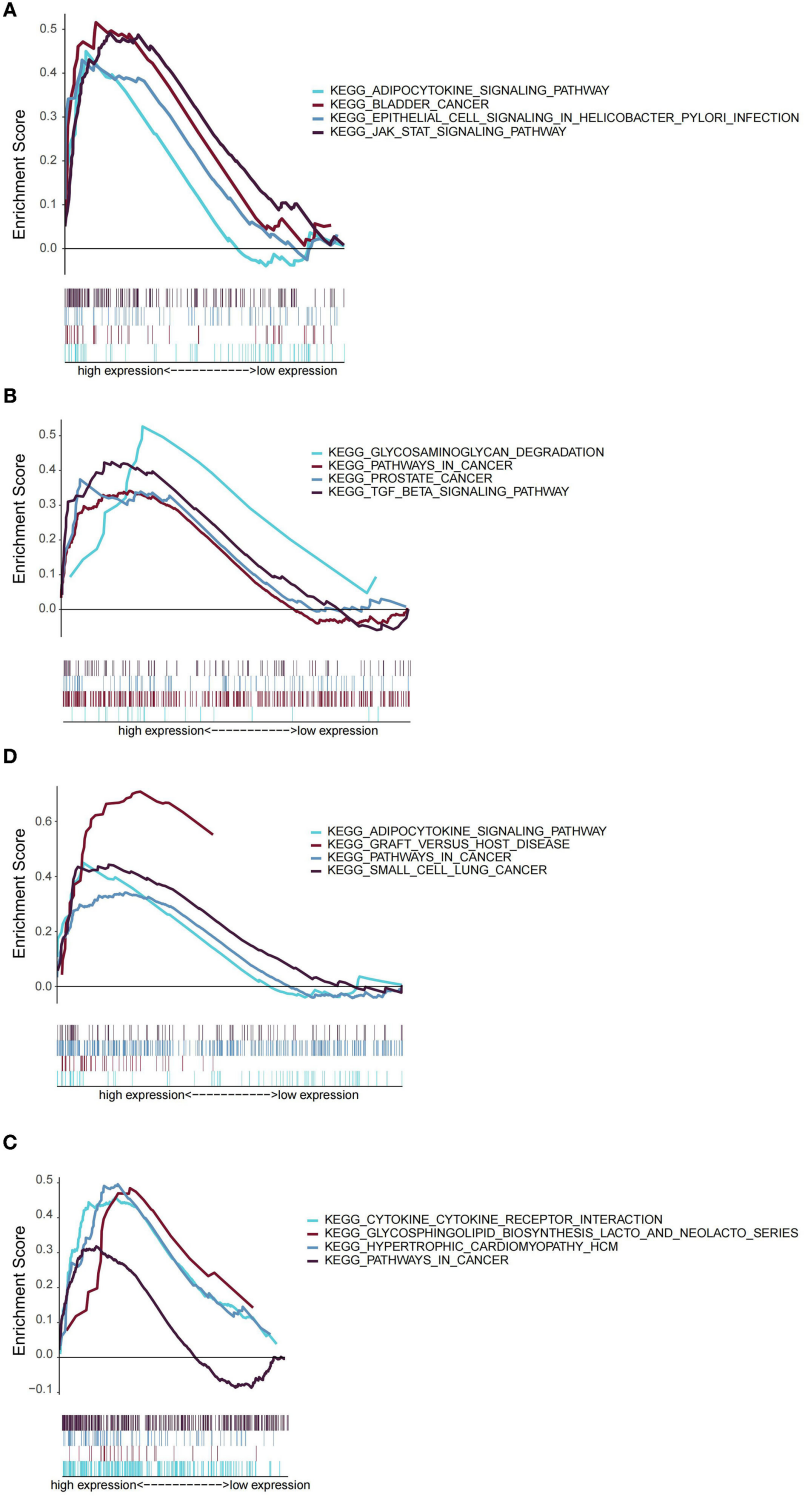
GSEA analysis of functions related to *AREG* **(A)**, *ATF3* **(B)**, *DUSP1* **(C)**, and *ZFP36* **(D)**.

Next, we explored the role of *AREG, ATF3, ZFP36*, and *DUSP1* in OSA by GSEA. According to the expression of each gene, OSA patients were divided into low- and high-expression groups. Interestingly, *AREG, ATF3, ZFP36*, and *DUSP1* were all associated with inflammation and cancer, specifically enrichment of adipocytokine signaling, bladder cancer, epithelial cell signaling in *Helicobacter pylori* infection, and JAK/STAT signaling pathways in patients with high *AREG* expression (Figure 7A). *ATF3* expression was related to glycosaminoglycan degradation, pathways in cancer, prostate cancer, and TGF-β signaling (Figure 7B), and *DUSP1* was associated with adipocytokine signaling, graft vs. host disease, pathways in cancer, and small cell lung cancer (Figure 7C). Finally, *ZFP36* was associated with cytokine-cytokine receptor interactions, glycosphingolipid biosynthesis (lacto and neolecto series), hypertrophic cardiomyopathy, and pathways in cancer (Figure 7D). *REG, ATF3, ZFP36*, and *DUSP1* therefore have both common and distinct roles in OSA.

## Discussion

The diagnostic value of hypoxia or its surrogates in OSA remains unclear. Here we investigated the diagnostic value of hypoxia-related genes in OSA and analyzed potential mechanisms by which the identified key hypoxia-related genes might be involved in OSA pathobiology.

Sixty-three genes were differentially expressed between control and OSA samples, which were significantly enriched in hypoxia-related pathways such as oxidative stress. Oxidative stress refers to an imbalance between anti-oxidants and pro-oxidants, resulting in the disruption of redox signaling and/or molecular damage as a consequence of ROS and reactive nitrogen species (RNS) overproduction (16). In OSA, the upper airways of patients collapse repeatedly during sleep, resulting in intermittent hypoxia and reoxygenation (17). This hypoxia/reoxygenation process is an important mechanism of target organ injury in OSA and it produces a large amount of ROS, leading to oxidative stress (18). Our results further demonstrate an important role for hypoxia in OSA. In addition, the identified genes were significantly enriched in inflammation-related signaling pathways such as IL-17 signaling, TNF signaling, and MAPK signaling. IL-17 is mainly secreted by Th17 cells, where it binds to cell surface receptors to activate downstream cellular signal transduction pathways and induce the secretion of pro-inflammatory cytokines (19). IL-17 also participates in the proliferation of neutrophils and dendritic cells (DC), immune cell maturation and chemotaxis, and synergizes with some cytokines to activate T cells, thereby amplifying the immune response and inflammatory destruction of target organs and contributing to several autoimmune and infectious diseases (20). In their analysis of adenoid hypertrophy of children with OSA, Sade et al. found a negative correlation between Th17/T cell subsets and OSA symptom severity (21). During long-term chronic hypoxia in adult OSA, Th17/Tregs become imbalanced, and Th17 and associated inflammatory cytokines may be involved in the occurrence and development of OSA (22). Therefore, IL-17 signaling may play an important role in the pathogenesis of OSA and its complication.

TNF-α plays an important role in sleep regulation, and excessive daytime drowsiness, one of the main consequences of OSA, is accompanied by an increase in TNF-α levels (23–25). In addition, TNF-α is a crucial player in immune and inflammatory responses (26), which may be involved in IH-driven OSA-related cardio-metabolic disease (27, 28). The MAPK signaling pathway plays an important role in central injury and regulatory mechanisms induced by CIH (29). Activated by hypoxia and other stimuli, MAPK enters the nucleus to regulate transcription and participate in cell proliferation, development, apoptosis, as well as the recognition, transmission, and amplification of a variety of biochemical response signals between cells (30), indicating that MAPK may mediate the OSA-related changes in cellular physiology caused by IH.

By intersecting DEGs with hypoxia-related genes, we obtained 17 hypoxia-related genes. Among them, we found that the expression of IL6 was significantly down-regulated in OSA samples compared with control samples, which is inconsistent with previous finding in OSA (31). According to the BMI in GSE135917, both OSA and control samples were obese and under chronic inflammation, which may affect the expressions of IL6. Besides, we also found that some of OSA and control individuals were taking medicines, such as duloxetine, insulin and vitamin D, which may either elevate or reduce the expression of IL6 (32–34). Those characteristics of OSA and control samples used in the current study may cause the conflicted results with previous finding. We subsequently constructed a diagnostic model that accurately distinguished between OSA and control samples based on four hypoxia-related genes: *AREG, ATF3, ZFP36*, and *DUSP1*. AREG, a member of the epidermal growth factor family (35), is an autocrine growth factor and mitogen for astrocytes, Schwann cells, and fibroblasts (36). It has been reported that hypoxia promotes AREG expression in intestinal epithelial cells in a cAMP response element-dependent manner (37). Furthermore, Yoji et al. detected significant increases in *AREG* transcript in intermittent hypoxia-treated rat vascular smooth muscle cells (38). Its role in OSA remains unknown but, tor the first time, our study found that *AREG1* is a potential diagnostic biomarker with high accuracy (AUC 0.856).

The expression of *ATF3*, a member of the CREB/ATF family, can be induced by hypoxia (39, 40), and numerous studies have reported its important role in hypoxia-related diseases (41, 42) but not in OSA. Jiang et al. demonstrated that hypoxia or ATF3 overexpression impaired mitochondrial function in differentiated 3T3-L1 fibroblast-like cells, and *ATF3* deletion partially recovered hypoxia-mediated mitochondrial dysfunction, suggesting that ATF3 may play a role in adipocyte hypoxia-mediated mitochondrial dysfunction in obesity (39). However, its exact role in OSA requires further *in vivo* and *in vitro* investigation. ZFP36 plays a significant role in regulating immune responses and inflammatory diseases by inhibiting the production of various inflammatory cytokines such as TNF-α in macrophages (43). Recent data have indicated that inflammation may play an important role in OSA (44). Zhang et al. found that the genotype distribution of single nucleotide polymorphisms rs251864 and rs17879933 in *ZFP36* were significantly different between moderate-to-severe OSA patients and control controls, but they were not directly associated with sleep apnea parameters, suggesting that ATF may be involved in regulating OSA in cooperation with other factors (45). It has also been reported that overnight IH in patients with OSA induces expression of *DUSP1*, which may mediate increases in manganese superoxide dismutase (MnSOD) expression and activity, contributing significantly to neutralizing the effects of the ROS elicited by OSA (46). Collectively, *AREG, ATF3, ZFP36*, and *DUSP1* are promising biomarkers for the diagnosis of OSA and are possibly functional in OSA pathogenesis. However, the detailed mechanisms of how they participate in OSA need to be established in future studies.

Finally, to obtain further insights into potentially relevant mechanisms in OSA pathogenesis, we used GSEA to analyze the signaling pathways related to *AREG, ATF3, ZFP36*, and *DUSP1*, and found that all four markers were related to inflammation and cancer-related pathways. For example, *AREG* and *DUSP2* were associated with adipocytokine signaling. There is increasing evidence that IH mediates at least some of its detrimental effects through adipose tissue inflammation and dysfunction (47), and adipocytokines secreted by adipocytes such as leptin, chemerin, resistin, adiponectin, and omentin 1 are closely associated with OSA (48). ATF3 was related to TGF-β signaling, which has been reported to be necessary for nuclear accumulation of the ATF3/cJun transcription complex and induction of pro-inflammatory responses (49). Compared with control subjects, Lin et al. found that OSA patients had higher TGF-β concentrations in exhaled breath condensates. These reports suggest that ATF3 may function in OSA *via* TGF-β signaling-mediated inflammation. Similarly, cytokine-cytokine interactions were significantly enriched in the high ZPF36-expressing group. Elevated levels of several cytokines, such as IL6 and TNF-α, are a common feature of OSA (50), suggesting that their interactions may also be important in OSA. Our results also reinforce the hypothesis that OSA may be considered a systemic inflammatory disease, with an imbalance in pro/anti-inflammatory cytokines contributing to its development and the progression of comorbidities. Of note, *AREG, ATF3, DUSP1*, and *ZFP36* were all related to cancers or pathways in cancer, and numerous studies have reported a role for these genes in different tumors (51–54). OSA is a recognized risk factor for cancer, mainly through hypoxia (55), but the exact mechanisms remain unclear. Combined with findings from our current study, we speculate that *AREG, ATF3, DUSP1*, and *ZFP36* may serve as a bridge between cancer and OSA.

This study helps to clarify the relationship between OSA hypopnea syndrome and hypoxia genes. However, it also has some limitations. First, this research is based on a retrospective analysis of existing databases, so prospective studies are needed to verify the *in silico* results. Second, our goal was to find a practical diagnostic model, but exactly how to use and measure these biomarkers in clinical practice requires further exploration. And it is worth mentioning that the OSA samples used in the current study were obese, and obesity is closely related to hypoxia. Therefore, the relationships between hypoxia-related genes in the diagnostic model and OSA may be due to obesity. Further collecting samples from subgroups of lean and obese OSA patients are needed to validate the clinical use of the current diagnostic model. Third, although OSA and related hypoxia genes were identified, their exact biological functions in OSA require direct expenormalrimental verification.

In conclusion, this study explored the diagnostic value of hypoxia-related genes in OSA, constructed a diagnostic model that can help to distinguish OSA from subjects, and unveiled potential molecular mechanisms of key hypoxia-related genes in OSA. Our findings may pave the way for accurately diagnosing OSA without the need for PSG.

## Data Availability Statement

The datasets presented in this study can be found in online repositories. The names of the repository/repositories and accession number(s) can be found at: the OSA-related data employed in this research were obtained from the GEO database GSE135917 (https://www.ncbi.nlm.nih.gov/geo/). Hypoxia-related genes were obtained from HALLMARK_HYPOXIA from the MSigDB database (http://www.gsea-msigdb.org/gsea/msigdb/cards/HALLMARK_HYPOXIA.html). The PPI network was from the STRING database (www.string-db.org/).

## Author Contributions

KH supervised and conceptualized the study. XW and ZP performed most of the bioinformatics analysis. WL, SZ, and QZ participated in the bioinformatics and statistical analyses. XW and KH wrote the manuscript. All authors read and approved the final manuscript.

## Funding

This study was supported by the National Natural Science Foundation of China (No. 81970082).

## Conflict of Interest

The authors declare that the research was conducted in the absence of any commercial or financial relationships that could be construed as a potential conflict of interest.

## Publisher's Note

All claims expressed in this article are solely those of the authors and do not necessarily represent those of their affiliated organizations, or those of the publisher, the editors and the reviewers. Any product that may be evaluated in this article, or claim that may be made by its manufacturer, is not guaranteed or endorsed by the publisher.

## Supplementary Material

Supplementary Figure 1 The expressions of hypoxia-related DEGs in OSA and control subjects.

Supplementary Table 1 Identified DEGs between OSA and control subjects in GSE135917.

Supplementary Table 2 GO enrichment analysis of DEGs identified in GSE135917.

Supplementary Table 3 KEGG pathway enrichment analysis of DEGs identified in GSE135917.
