# Supplementary figures and images for: The Discovery, Validation, and Function of Hypoxia-Related Gene Biomarkers for Obstructive Sleep Apnea
Source: Front Med (Lausanne). 2022 Mar 17;9:813459. doi: 10.3389/fmed.2022.813459 (PMC8970318; doi:10.3389/fmed.2022.813459)

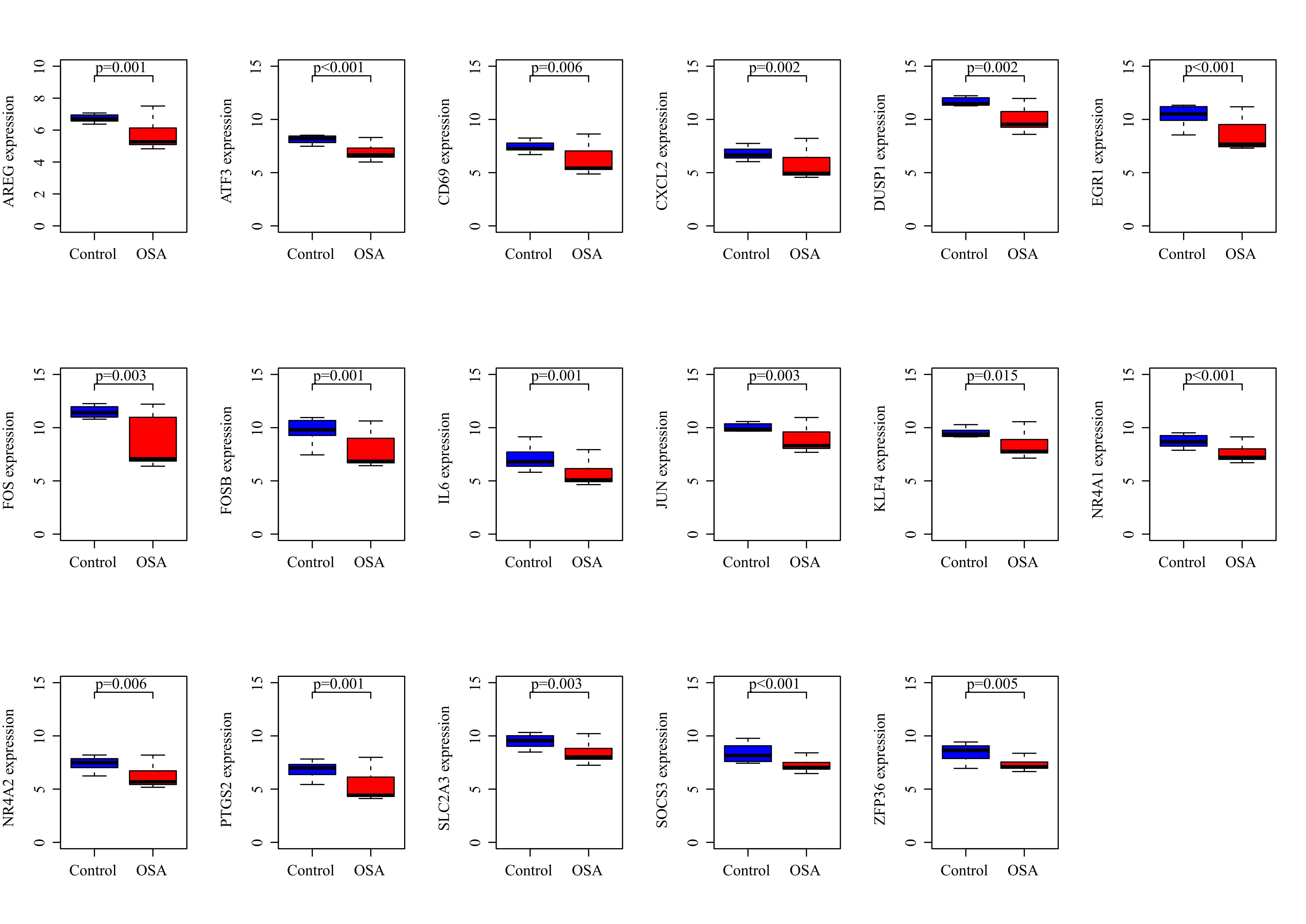

Supplement: Supplementary Figure 1 — The expressions of hypoxia-related DEGs in OSA and control subjects. [file Image_1.JPEG]
