# Supplementary material for: The Discovery, Validation, and Function of Hypoxia-Related Gene Biomarkers for Obstructive Sleep Apnea
Source: Front Med (Lausanne). 2022 Mar 17;9:813459. doi: 10.3389/fmed.2022.813459 (PMC8970318; doi:10.3389/fmed.2022.813459)
Supplement: Supplementary Table 1 — Identified DEGs between OSA and control subjects in GSE135917. [file Table_1.DOCX]

Supplementary Table 1. Identified DEGs between OSA and non-OSA subjects in GSE135917

| id | logFC | AveExpr | t | P.Value | adj.P.Val | B |
| --- | --- | --- | --- | --- | --- | --- |
| FOS | -3.019654309 | 8.85492069 | -4.10804825 | 0.000161936 | 0.000998696 | 0.735253057 |
| FOSB | -2.055925853 | 8.071574071 | -3.903377878 | 0.000307434 | 0.001551887 | 0.138448727 |
| EGR1 | -1.968809184 | 8.800033714 | -4.14661471 | 0.00014331 | 0.000920616 | 0.849249643 |
| MIR21 | -1.781952493 | 7.639361905 | -4.36295391 | 7.17E-05 | 0.00059813 | 1.496976836 |
| PTGS2 | -1.704739279 | 5.444940714 | -4.117314033 | 0.000157257 | 0.000979432 | 0.762598575 |
| DUSP1 | -1.529402809 | 10.27747326 | -4.212241383 | 0.000116296 | 0.000810769 | 1.044288004 |
| IL6 | -1.472338632 | 5.901968571 | -3.692602053 | 0.000586618 | 0.002460271 | -0.460506552 |
| PKHD1L1 | -1.381483375 | 4.54531019 | -3.800835588 | 0.000421776 | 0.00193213 | -0.155038155 |
| LINC00917 | -1.355052051 | 5.828149476 | -4.531720066 | 4.14E-05 | 0.000435465 | 2.011178526 |
| CD69 | -1.35421275 | 6.315500286 | -3.469127366 | 0.001143727 | 0.004049174 | -1.076139626 |
| CXCL2 | -1.338358691 | 5.699185524 | -3.893673959 | 0.00031682 | 0.001585095 | 0.11051098 |
| ITLN1 | -1.315907206 | 5.397059286 | -3.034685081 | 0.003950502 | 0.010559821 | -2.207186588 |
| MOP-1 | -1.315007875 | 5.26487169 | -2.908099388 | 0.005578088 | 0.013987832 | -2.518420349 |
| CYR61 | -1.28448725 | 7.606123238 | -3.555512071 | 0.000885538 | 0.003319665 | -0.84067372 |
| NFKBIZ | -1.259354081 | 7.268307762 | -3.522013143 | 0.000978241 | 0.003591506 | -0.932370227 |
| PDLIM3 | -1.239652404 | 6.548032738 | -5.114787038 | 5.95E-06 | 0.000159897 | 3.835826641 |
| DSC3 | -1.23627725 | 4.128215381 | -4.900723139 | 1.22E-05 | 0.0002246 | 3.158396755 |
| NR4A2 | -1.230650294 | 6.402016286 | -3.465118917 | 0.001157305 | 0.004083569 | -1.086986181 |
| RNVU1-19 | -1.229743368 | 4.964022714 | -5.405474512 | 2.22E-06 | 0.000102826 | 4.766572299 |
| RN7SL600P | -1.225936243 | 7.461417048 | -3.149308514 | 0.002871641 | 0.008210718 | -1.917911156 |
| CLDN1 | -1.20593075 | 5.187219595 | -3.398981307 | 0.001404778 | 0.004727154 | -1.264904838 |
| AREG | -1.197820331 | 5.886899048 | -4.149855441 | 0.000141844 | 0.000915738 | 0.858849933 |
| RGS1 | -1.194428449 | 5.538035357 | -3.601559606 | 0.000771737 | 0.003003154 | -0.713847261 |
| KLHL4 | -1.157816574 | 4.509032667 | -4.643871567 | 2.87E-05 | 0.000352748 | 2.356749004 |
| NR4A1 | -1.141645051 | 7.800744024 | -3.977317529 | 0.000244239 | 0.00131994 | 0.352413168 |
| CNTN4 | -1.130789618 | 4.689159381 | -5.381083925 | 2.41E-06 | 0.000106376 | 4.688069256 |
| ATF3 | -1.126625978 | 7.204417071 | -4.045746755 | 0.000197093 | 0.001138401 | 0.55210232 |
| JUN | -1.118465706 | 8.969080881 | -3.380055688 | 0.001484391 | 0.004940764 | -1.315449655 |
| VN1R17P | -1.113491515 | 4.325636357 | -5.24553674 | 3.83E-06 | 0.000128467 | 4.253095927 |
| OR52N3P | -1.11103686 | 4.690919833 | -5.811164199 | 5.53E-07 | 6.81E-05 | 6.080660038 |
| CSRNP1 | -1.103651441 | 7.313168381 | -5.095753182 | 6.35E-06 | 0.000164775 | 3.775290566 |
| KLF4 | -1.100357125 | 8.428507333 | -3.252167128 | 0.002145955 | 0.006534333 | -1.652575021 |
| GPR1 | -1.096812191 | 4.825134167 | -4.896753731 | 1.24E-05 | 0.000226135 | 3.145909582 |
| LOC102724208 | -1.082770419 | 4.78085419 | -6.124239234 | 1.87E-07 | 4.15E-05 | 7.10224648 |
| SLC2A3 | -1.077773949 | 8.625471452 | -3.214372332 | 0.002389695 | 0.007102045 | -1.750687577 |
| CCL18 | -1.074498338 | 7.391054262 | -2.829481345 | 0.006883565 | 0.016593749 | -2.707180452 |
| SLPI | -1.070085206 | 6.586051548 | -3.858930213 | 0.000352747 | 0.001708818 | 0.010761495 |
| RNU6-951P | -1.068938353 | 3.833748452 | -6.798716093 | 1.81E-08 | 2.35E-05 | 9.309416393 |
| EPPIN | -1.068383794 | 4.806712881 | -5.936028194 | 3.59E-07 | 5.54E-05 | 6.487549352 |
| RNA5SP494 | -1.066310221 | 5.034755738 | -4.146338504 | 0.000143436 | 0.000920915 | 0.848431565 |
| ZFP36 | -1.062196912 | 7.58082981 | -4.010665324 | 0.000220039 | 0.001225969 | 0.449531623 |
| SCN7A | -1.049018551 | 4.385638881 | -5.364706819 | 2.55E-06 | 0.000108111 | 4.635396406 |
| RNU6-1014P | -1.048692728 | 4.216598643 | -5.327247441 | 2.90E-06 | 0.000112205 | 4.515036445 |
| SULF1 | -1.048074279 | 7.434755667 | -3.688999742 | 0.000593053 | 0.002479227 | -0.470594431 |
| SOCS3 | -1.038889279 | 7.515594143 | -3.721113372 | 0.000538005 | 0.002299843 | -0.380481812 |
| ZNF98 | -1.036495581 | 4.629315024 | -3.898811931 | 0.000311816 | 0.001567778 | 0.12529909 |
| OR7E4P | -1.028493257 | 3.920215595 | -5.388176002 | 2.36E-06 | 0.000105281 | 4.71088872 |
| SAA2-SAA4 | -1.021880559 | 6.165231357 | -4.263787585 | 9.86E-05 | 0.000728038 | 1.198383822 |
| SNORD115-32 | -1.021654147 | 4.290812143 | -6.974595164 | 9.87E-09 | 2.20E-05 | 9.883846154 |
| APOB | -1.009967618 | 6.668800167 | -4.240673125 | 0.000106196 | 0.000762202 | 1.129187396 |
| WT1 | -1.005139882 | 6.315171333 | -4.255099613 | 0.000101403 | 0.000739224 | 1.172356911 |
| OR10K2 | -1.000141147 | 4.659101429 | -5.197762556 | 4.50E-06 | 0.000137467 | 4.100351867 |
| PLA2G7 | 1.024015243 | 8.272829595 | 2.676662608 | 0.010265705 | 0.023076053 | -3.063687325 |
| TM4SF19 | 1.092366221 | 8.545210714 | 2.763409495 | 0.008194264 | 0.019137741 | -2.863034589 |
| ERAP2 | 1.122354324 | 7.520103048 | 3.190852879 | 0.002554343 | 0.007491068 | -1.811383081 |
| USP9Y | 1.123491044 | 5.944396 | 2.559722377 | 0.013821412 | 0.029468651 | -3.326810712 |
| UTY | 1.289378691 | 6.359968 | 2.593050031 | 0.012707864 | 0.027496934 | -3.252700629 |
| HBA2 | 1.291037294 | 11.42167893 | 3.3898314 | 0.001442747 | 0.004832518 | -1.289362231 |
| IMPAD1 | 1.32385564 | 9.079682786 | 4.545670697 | 3.96E-05 | 0.000424752 | 2.054003431 |
| PPBP | 1.63601139 | 7.285542548 | 2.954708586 | 0.004917108 | 0.012632067 | -2.404854433 |
| EIF1AY | 1.691052963 | 6.693357262 | 2.959615065 | 0.004851954 | 0.012489468 | -2.39282891 |
| HBB | 1.839369162 | 9.856880881 | 3.565623304 | 0.000859251 | 0.003250924 | -0.812901746 |
| EGFL6 | 2.410422309 | 9.6017375 | 4.499259818 | 4.60E-05 | 0.000463923 | 1.911716871 |
