# Supplementary material for: The Discovery, Validation, and Function of Hypoxia-Related Gene Biomarkers for Obstructive Sleep Apnea
Source: Front Med (Lausanne). 2022 Mar 17;9:813459. doi: 10.3389/fmed.2022.813459 (PMC8970318; doi:10.3389/fmed.2022.813459)
Supplement: Supplementary Table 2 — GO enrichment analysis of DEGs identified in GSE135917. [file Table_2.DOCX]

Supplementary Table 2. GO enrichment analysis of DEGs identified in GSE135917

| ONTOLOGY | ID | Description | GeneRatio | BgRatio | pvalue | p.adjust | qvalue | geneID | Count |
| --- | --- | --- | --- | --- | --- | --- | --- | --- | --- |
| BP | GO:0045444 | fat cell differentiation | 6月16日 | 221/18862 | 1.76E-08 | 1.35E-05 | 6.37E-06 | PTGS2/ZFP36/NR4A1/IL6/NR4A2/KLF4 | 6 |
| BP | GO:1902895 | positive regulation of pri-miRNA transcription by RNA polymerase II | 4月16日 | 40/18862 | 3.10E-08 | 1.35E-05 | 6.37E-06 | EGR1/FOS/JUN/KLF4 | 4 |
| BP | GO:0006979 | response to oxidative stress | 7月16日 | 444/18862 | 3.64E-08 | 1.35E-05 | 6.37E-06 | DUSP1/PTGS2/FOS/IL6/NR4A2/JUN/KLF4 | 7 |
| BP | GO:0051384 | response to glucocorticoid | 5月16日 | 135/18862 | 7.15E-08 | 1.70E-05 | 7.98E-06 | DUSP1/FOS/ZFP36/FOSB/IL6 | 5 |
| BP | GO:1902893 | regulation of pri-miRNA transcription by RNA polymerase II | 4月16日 | 51/18862 | 8.42E-08 | 1.70E-05 | 7.98E-06 | EGR1/FOS/JUN/KLF4 | 4 |
| BP | GO:0061614 | pri-miRNA transcription by RNA polymerase II | 4月16日 | 52/18862 | 9.12E-08 | 1.70E-05 | 7.98E-06 | EGR1/FOS/JUN/KLF4 | 4 |
| BP | GO:0031960 | response to corticosteroid | 5月16日 | 152/18862 | 1.29E-07 | 2.06E-05 | 9.71E-06 | DUSP1/FOS/ZFP36/FOSB/IL6 | 5 |
| BP | GO:0000302 | response to reactive oxygen species | 5月16日 | 224/18862 | 8.87E-07 | 0.000119436 | 5.62E-05 | DUSP1/FOS/IL6/JUN/KLF4 | 5 |
| BP | GO:0051591 | response to cAMP | 4月16日 | 93/18862 | 9.63E-07 | 0.000119436 | 5.62E-05 | DUSP1/FOS/FOSB/JUN | 4 |
| BP | GO:0034599 | cellular response to oxidative stress | 5月16日 | 299/18862 | 3.66E-06 | 0.000373055 | 0.000175584 | FOS/IL6/NR4A2/JUN/KLF4 | 5 |
| BP | GO:0046683 | response to organophosphorus | 4月16日 | 130/18862 | 3.68E-06 | 0.000373055 | 0.000175584 | DUSP1/FOS/FOSB/JUN | 4 |
| BP | GO:0042542 | response to hydrogen peroxide | 4月16日 | 135/18862 | 4.27E-06 | 0.000397368 | 0.000187028 | DUSP1/IL6/JUN/KLF4 | 4 |
| BP | GO:0014074 | response to purine-containing compound | 4月16日 | 144/18862 | 5.52E-06 | 0.000441079 | 0.000207601 | DUSP1/FOS/FOSB/JUN | 4 |
| BP | GO:0032496 | response to lipopolysaccharide | 5月16日 | 326/18862 | 5.59E-06 | 0.000441079 | 0.000207601 | FOS/ZFP36/CXCL2/IL6/JUN | 5 |
| BP | GO:0048545 | response to steroid hormone | 5月16日 | 330/18862 | 5.93E-06 | 0.000441079 | 0.000207601 | DUSP1/FOS/ZFP36/FOSB/IL6 | 5 |
| BP | GO:0002237 | response to molecule of bacterial origin | 5月16日 | 346/18862 | 7.46E-06 | 0.000496889 | 0.000233869 | FOS/ZFP36/CXCL2/IL6/JUN | 5 |
| BP | GO:0062197 | cellular response to chemical stress | 5月16日 | 347/18862 | 7.57E-06 | 0.000496889 | 0.000233869 | FOS/IL6/NR4A2/JUN/KLF4 | 5 |
| BP | GO:0034614 | cellular response to reactive oxygen species | 4月16日 | 159/18862 | 8.18E-06 | 0.000506981 | 0.000238619 | FOS/IL6/JUN/KLF4 | 4 |
| BP | GO:1901654 | response to ketone | 4月16日 | 193/18862 | 1.76E-05 | 0.001031643 | 0.000485559 | DUSP1/FOS/FOSB/KLF4 | 4 |
| BP | GO:0035914 | skeletal muscle cell differentiation | 3月16日 | 64/18862 | 2.02E-05 | 0.001128073 | 0.000530946 | EGR1/FOS/ATF3 | 3 |
| BP | GO:0009991 | response to extracellular stimulus | 5月16日 | 477/18862 | 3.51E-05 | 0.001843764 | 0.000867797 | FOS/ATF3/ZFP36/NR4A2/JUN | 5 |
| BP | GO:0070373 | negative regulation of ERK1 and ERK2 cascade | 3月16日 | 78/18862 | 3.66E-05 | 0.001843764 | 0.000867797 | DUSP1/ATF3/KLF4 | 3 |
| BP | GO:0031668 | cellular response to extracellular stimulus | 4月16日 | 235/18862 | 3.80E-05 | 0.001843764 | 0.000867797 | FOS/ATF3/NR4A2/JUN | 4 |
| BP | GO:0032642 | regulation of chemokine production | 3月16日 | 89/18862 | 5.44E-05 | 0.002529403 | 0.001190504 | EGR1/IL6/KLF4 | 3 |
| BP | GO:1901216 | positive regulation of neuron death | 3月16日 | 92/18862 | 6.01E-05 | 0.002662424 | 0.001253112 | EGR1/FOS/JUN | 3 |
| BP | GO:0002042 | cell migration involved in sprouting angiogenesis | 3月16日 | 93/18862 | 6.20E-05 | 0.002662424 | 0.001253112 | PTGS2/NR4A1/KLF4 | 3 |
| BP | GO:0032602 | chemokine production | 3月16日 | 95/18862 | 6.61E-05 | 0.002731867 | 0.001285797 | EGR1/IL6/KLF4 | 3 |
| BP | GO:0043618 | regulation of transcription from RNA polymerase II promoter in response to stress | 3月16日 | 109/18862 | 9.95E-05 | 0.003794848 | 0.001786105 | EGR1/ATF3/JUN | 3 |
| BP | GO:1901214 | regulation of neuron death | 4月16日 | 302/18862 | 0.000100717 | 0.003794848 | 0.001786105 | EGR1/FOS/NR4A2/JUN | 4 |
| BP | GO:0071496 | cellular response to external stimulus | 4月16日 | 303/18862 | 0.000102012 | 0.003794848 | 0.001786105 | FOS/ATF3/NR4A2/JUN | 4 |
| BP | GO:0060326 | cell chemotaxis | 4月16日 | 306/18862 | 0.00010597 | 0.003814927 | 0.001795556 | DUSP1/NR4A1/CXCL2/IL6 | 4 |
| BP | GO:0043620 | regulation of DNA-templated transcription in response to stress | 3月16日 | 115/18862 | 0.000116684 | 0.004069349 | 0.001915304 | EGR1/ATF3/JUN | 3 |
| BP | GO:0051412 | response to corticosterone | 2月16日 | 20/18862 | 0.000127041 | 0.004296292 | 0.002022118 | FOS/FOSB | 2 |
| BP | GO:0070997 | neuron death | 4月16日 | 342/18862 | 0.000162662 | 0.005339153 | 0.002512957 | EGR1/FOS/NR4A2/JUN | 4 |
| BP | GO:0010038 | response to metal ion | 4月16日 | 352/18862 | 0.000181695 | 0.005793474 | 0.002726791 | DUSP1/FOS/FOSB/JUN | 4 |
| BP | GO:0045598 | regulation of fat cell differentiation | 3月16日 | 136/18862 | 0.000191684 | 0.005942212 | 0.002796797 | PTGS2/ZFP36/IL6 | 3 |
| BP | GO:0032682 | negative regulation of chemokine production | 2月16日 | 25/18862 | 0.000200095 | 0.006035306 | 0.002840613 | IL6/KLF4 | 2 |
| BP | GO:0050727 | regulation of inflammatory response | 4月16日 | 366/18862 | 0.00021099 | 0.006196431 | 0.002916449 | PTGS2/SOCS3/IL6/KLF4 | 4 |
| BP | GO:0051592 | response to calcium ion | 3月16日 | 145/18862 | 0.000231554 | 0.00652525 | 0.003071213 | DUSP1/FOS/FOSB | 3 |
| BP | GO:0002675 | positive regulation of acute inflammatory response | 2月16日 | 27/18862 | 0.00023388 | 0.00652525 | 0.003071213 | PTGS2/IL6 | 2 |
| BP | GO:0007519 | skeletal muscle tissue development | 3月16日 | 147/18862 | 0.000241087 | 0.006562277 | 0.00308864 | EGR1/FOS/ATF3 | 3 |
| BP | GO:0010575 | positive regulation of vascular endothelial growth factor production | 2月16日 | 28/18862 | 0.000251746 | 0.006689254 | 0.003148404 | PTGS2/IL6 | 2 |
| BP | GO:2000637 | positive regulation of gene silencing by miRNA | 2月16日 | 29/18862 | 0.00027026 | 0.00688872 | 0.003242286 | ZFP36/IL6 | 2 |
| BP | GO:1901653 | cellular response to peptide | 4月16日 | 391/18862 | 0.000271598 | 0.00688872 | 0.003242286 | NR4A1/SOCS3/NR4A2/KLF4 | 4 |
| BP | GO:0060148 | positive regulation of posttranscriptional gene silencing | 2月16日 | 30/18862 | 0.000289421 | 0.006947203 | 0.003269811 | ZFP36/IL6 | 2 |
| BP | GO:0070102 | interleukin-6-mediated signaling pathway | 2月16日 | 30/18862 | 0.000289421 | 0.006947203 | 0.003269811 | SOCS3/IL6 | 2 |
| BP | GO:0060538 | skeletal muscle organ development | 3月16日 | 157/18862 | 0.000292579 | 0.006947203 | 0.003269811 | EGR1/FOS/ATF3 | 3 |
| BP | GO:0048660 | regulation of smooth muscle cell proliferation | 3月16日 | 160/18862 | 0.000309305 | 0.007191348 | 0.003384722 | IL6/JUN/KLF4 | 3 |
| BP | GO:0048659 | smooth muscle cell proliferation | 3月16日 | 162/18862 | 0.000320793 | 0.007306215 | 0.003438786 | IL6/JUN/KLF4 | 3 |
| BP | GO:0051385 | response to mineralocorticoid | 2月16日 | 32/18862 | 0.000329681 | 0.00735847 | 0.003463381 | FOS/FOSB | 2 |
| BP | GO:0050673 | epithelial cell proliferation | 4月16日 | 428/18862 | 0.000383105 | 0.00838325 | 0.00394571 | AREG/ZFP36/NR4A1/IL6 | 4 |
| BP | GO:0043534 | blood vessel endothelial cell migration | 3月16日 | 175/18862 | 0.000402229 | 0.008523391 | 0.00401167 | PTGS2/NR4A1/KLF4 | 3 |
| BP | GO:0043434 | response to peptide hormone | 4月16日 | 435/18862 | 0.000407414 | 0.008523391 | 0.00401167 | EGR1/NR4A1/SOCS3/NR4A2 | 4 |
| BP | GO:0051090 | regulation of DNA-binding transcription factor activity | 4月16日 | 444/18862 | 0.000440275 | 0.008523391 | 0.00401167 | FOS/IL6/JUN/KLF4 | 4 |
| BP | GO:0010837 | regulation of keratinocyte proliferation | 2月16日 | 37/18862 | 0.000441582 | 0.008523391 | 0.00401167 | AREG/ZFP36 | 2 |
| BP | GO:1904994 | regulation of leukocyte adhesion to vascular endothelial cell | 2月16日 | 37/18862 | 0.000441582 | 0.008523391 | 0.00401167 | IL6/KLF4 | 2 |
| BP | GO:0043409 | negative regulation of MAPK cascade | 3月16日 | 181/18862 | 0.00044391 | 0.008523391 | 0.00401167 | DUSP1/ATF3/KLF4 | 3 |
| BP | GO:0009314 | response to radiation | 4月16日 | 447/18862 | 0.000451639 | 0.008523391 | 0.00401167 | DUSP1/EGR1/FOS/JUN | 4 |
| BP | GO:0002040 | sprouting angiogenesis | 3月16日 | 184/18862 | 0.000465754 | 0.008523391 | 0.00401167 | PTGS2/NR4A1/KLF4 | 3 |
| BP | GO:0045429 | positive regulation of nitric oxide biosynthetic process | 2月16日 | 38/18862 | 0.000465884 | 0.008523391 | 0.00401167 | PTGS2/KLF4 | 2 |
| BP | GO:0071276 | cellular response to cadmium ion | 2月16日 | 38/18862 | 0.000465884 | 0.008523391 | 0.00401167 | FOS/JUN | 2 |
| BP | GO:1904407 | positive regulation of nitric oxide metabolic process | 2月16日 | 39/18862 | 0.000490824 | 0.008782892 | 0.004133808 | PTGS2/KLF4 | 2 |
| BP | GO:0060759 | regulation of response to cytokine stimulus | 3月16日 | 189/18862 | 0.000503678 | 0.008782892 | 0.004133808 | SOCS3/IL6/KLF4 | 3 |
| BP | GO:0071248 | cellular response to metal ion | 3月16日 | 189/18862 | 0.000503678 | 0.008782892 | 0.004133808 | FOS/FOSB/JUN | 3 |
| BP | GO:0150077 | regulation of neuroinflammatory response | 2月16日 | 40/18862 | 0.000516402 | 0.008866221 | 0.004173028 | PTGS2/IL6 | 2 |
| BP | GO:0050731 | positive regulation of peptidyl-tyrosine phosphorylation | 3月16日 | 193/18862 | 0.000535407 | 0.009053253 | 0.004261058 | AREG/SOCS3/IL6 | 3 |
| BP | GO:0042594 | response to starvation | 3月16日 | 196/18862 | 0.000560029 | 0.009328238 | 0.004390484 | ATF3/ZFP36/JUN | 3 |
| BP | GO:0071222 | cellular response to lipopolysaccharide | 3月16日 | 197/18862 | 0.000568394 | 0.009328356 | 0.004390539 | ZFP36/CXCL2/IL6 | 3 |
| BP | GO:0009612 | response to mechanical stimulus | 3月16日 | 202/18862 | 0.000611429 | 0.009889195 | 0.004654507 | FOS/FOSB/JUN | 3 |
| BP | GO:0150076 | neuroinflammatory response | 2月16日 | 44/18862 | 0.000625065 | 0.009965321 | 0.004690337 | PTGS2/IL6 | 2 |
| BP | GO:0032570 | response to progesterone | 2月16日 | 45/18862 | 0.000653814 | 0.010276856 | 0.004836966 | FOS/FOSB | 2 |
| BP | GO:0071219 | cellular response to molecule of bacterial origin | 3月16日 | 209/18862 | 0.000675109 | 0.010303322 | 0.004849423 | ZFP36/CXCL2/IL6 | 3 |
| BP | GO:0006953 | acute-phase response | 2月16日 | 46/18862 | 0.000683195 | 0.010303322 | 0.004849423 | PTGS2/IL6 | 2 |
| BP | GO:0071354 | cellular response to interleukin-6 | 2月16日 | 46/18862 | 0.000683195 | 0.010303322 | 0.004849423 | SOCS3/IL6 | 2 |
| BP | GO:0071241 | cellular response to inorganic substance | 3月16日 | 216/18862 | 0.000742892 | 0.010780962 | 0.005074231 | FOS/FOSB/JUN | 3 |
| BP | GO:0002673 | regulation of acute inflammatory response | 2月16日 | 48/18862 | 0.000743848 | 0.010780962 | 0.005074231 | PTGS2/IL6 | 2 |
| BP | GO:0043616 | keratinocyte proliferation | 2月16日 | 48/18862 | 0.000743848 | 0.010780962 | 0.005074231 | AREG/ZFP36 | 2 |
| BP | GO:0097529 | myeloid leukocyte migration | 3月16日 | 218/18862 | 0.000763027 | 0.010917151 | 0.005138331 | DUSP1/CXCL2/IL6 | 3 |
| BP | GO:0038066 | p38MAPK cascade | 2月16日 | 49/18862 | 0.000775117 | 0.01094976 | 0.005153679 | DUSP1/ZFP36 | 2 |
| BP | GO:0033002 | muscle cell proliferation | 3月16日 | 222/18862 | 0.000804337 | 0.011118871 | 0.005233274 | IL6/JUN/KLF4 | 3 |
| BP | GO:0070741 | response to interleukin-6 | 2月16日 | 50/18862 | 0.000807015 | 0.011118871 | 0.005233274 | SOCS3/IL6 | 2 |
| BP | GO:0030595 | leukocyte chemotaxis | 3月16日 | 226/18862 | 0.00084705 | 0.011528149 | 0.005425907 | DUSP1/CXCL2/IL6 | 3 |
| BP | GO:0071216 | cellular response to biotic stimulus | 3月16日 | 233/18862 | 0.000925234 | 0.012292393 | 0.00578561 | ZFP36/CXCL2/IL6 | 3 |
| BP | GO:0097305 | response to alcohol | 3月16日 | 233/18862 | 0.000925234 | 0.012292393 | 0.00578561 | FOS/FOSB/KLF4 | 3 |
| BP | GO:0061756 | leukocyte adhesion to vascular endothelial cell | 2月16日 | 54/18862 | 0.000940862 | 0.01235297 | 0.005814122 | IL6/KLF4 | 2 |
| BP | GO:1903428 | positive regulation of reactive oxygen species biosynthetic process | 2月16日 | 55/18862 | 0.000975884 | 0.012663798 | 0.005960418 | PTGS2/KLF4 | 2 |
| BP | GO:0032731 | positive regulation of interleukin-1 beta production | 2月16日 | 56/18862 | 0.001011528 | 0.012975459 | 0.006107106 | EGR1/IL6 | 2 |
| BP | GO:0043551 | regulation of phosphatidylinositol 3-kinase activity | 2月16日 | 57/18862 | 0.001047792 | 0.013287912 | 0.006254167 | SOCS3/KLF4 | 2 |
| BP | GO:0002763 | positive regulation of myeloid leukocyte differentiation | 2月16日 | 58/18862 | 0.001084677 | 0.013449996 | 0.006330455 | FOS/JUN | 2 |
| BP | GO:0010574 | regulation of vascular endothelial growth factor production | 2月16日 | 58/18862 | 0.001084677 | 0.013449996 | 0.006330455 | PTGS2/IL6 | 2 |
| BP | GO:0006352 | DNA-templated transcription, initiation | 3月16日 | 249/18862 | 0.001120842 | 0.013612544 | 0.006406961 | NR4A1/NR4A2/JUN | 3 |
| BP | GO:0045428 | regulation of nitric oxide biosynthetic process | 2月16日 | 59/18862 | 0.001122181 | 0.013612544 | 0.006406961 | PTGS2/KLF4 | 2 |
| BP | GO:0032722 | positive regulation of chemokine production | 2月16日 | 60/18862 | 0.001160303 | 0.013923639 | 0.006553382 | EGR1/IL6 | 2 |
| BP | GO:0046686 | response to cadmium ion | 2月16日 | 61/18862 | 0.001199043 | 0.014085596 | 0.00662961 | FOS/JUN | 2 |
| BP | GO:0080164 | regulation of nitric oxide metabolic process | 2月16日 | 61/18862 | 0.001199043 | 0.014085596 | 0.00662961 | PTGS2/KLF4 | 2 |
| BP | GO:0010573 | vascular endothelial growth factor production | 2月16日 | 62/18862 | 0.001238398 | 0.014284227 | 0.006723099 | PTGS2/IL6 | 2 |
| BP | GO:0045637 | regulation of myeloid cell differentiation | 3月16日 | 258/18862 | 0.00124155 | 0.014284227 | 0.006723099 | FOS/ZFP36/JUN | 3 |
| BP | GO:0050730 | regulation of peptidyl-tyrosine phosphorylation | 3月16日 | 262/18862 | 0.001297737 | 0.014778311 | 0.006955647 | AREG/SOCS3/IL6 | 3 |
| BP | GO:0032732 | positive regulation of interleukin-1 production | 2月16日 | 64/18862 | 0.001318955 | 0.014868221 | 0.006997965 | EGR1/IL6 | 2 |
| BP | GO:0045600 | positive regulation of fat cell differentiation | 2月16日 | 66/18862 | 0.001401966 | 0.015645939 | 0.00736401 | PTGS2/ZFP36 | 2 |
| BP | GO:0002548 | monocyte chemotaxis | 2月16日 | 68/18862 | 0.001487423 | 0.01643529 | 0.007735531 | DUSP1/IL6 | 2 |
| BP | GO:0042531 | positive regulation of tyrosine phosphorylation of STAT protein | 2月16日 | 69/18862 | 0.001531067 | 0.016509581 | 0.007770497 | SOCS3/IL6 | 2 |
| BP | GO:0043550 | regulation of lipid kinase activity | 2月16日 | 69/18862 | 0.001531067 | 0.016509581 | 0.007770497 | SOCS3/KLF4 | 2 |
| BP | GO:0043542 | endothelial cell migration | 3月16日 | 278/18862 | 0.001538527 | 0.016509581 | 0.007770497 | PTGS2/NR4A1/KLF4 | 3 |
| BP | GO:0006809 | nitric oxide biosynthetic process | 2月16日 | 73/18862 | 0.001711721 | 0.018193148 | 0.008562895 | PTGS2/KLF4 | 2 |
| BP | GO:0051348 | negative regulation of transferase activity | 3月16日 | 296/18862 | 0.001841121 | 0.019153539 | 0.009014918 | DUSP1/ZFP36/SOCS3 | 3 |
| BP | GO:0009791 | post-embryonic development | 2月16日 | 76/18862 | 0.001853568 | 0.019153539 | 0.009014918 | NR4A2/KLF4 | 2 |
| BP | GO:0060395 | SMAD protein signal transduction | 2月16日 | 76/18862 | 0.001853568 | 0.019153539 | 0.009014918 | FOS/JUN | 2 |
| BP | GO:0046209 | nitric oxide metabolic process | 2月16日 | 77/18862 | 0.001902057 | 0.019297229 | 0.009082548 | PTGS2/KLF4 | 2 |
| BP | GO:0090049 | regulation of cell migration involved in sprouting angiogenesis | 2月16日 | 77/18862 | 0.001902057 | 0.019297229 | 0.009082548 | PTGS2/KLF4 | 2 |
| BP | GO:0070372 | regulation of ERK1 and ERK2 cascade | 3月16日 | 301/18862 | 0.001931323 | 0.019417627 | 0.009139215 | DUSP1/ATF3/KLF4 | 3 |
| BP | GO:2001057 | reactive nitrogen species metabolic process | 2月16日 | 78/18862 | 0.001951146 | 0.01944178 | 0.009150583 | PTGS2/KLF4 | 2 |
| BP | GO:1990830 | cellular response to leukemia inhibitory factor | 2月16日 | 79/18862 | 0.002000837 | 0.019760474 | 0.009300581 | SOCS3/KLF4 | 2 |
| BP | GO:1990823 | response to leukemia inhibitory factor | 2月16日 | 80/18862 | 0.002051126 | 0.020079446 | 0.009450711 | SOCS3/KLF4 | 2 |
| BP | GO:0060761 | negative regulation of response to cytokine stimulus | 2月16日 | 81/18862 | 0.002102015 | 0.020398681 | 0.009600964 | IL6/KLF4 | 2 |
| BP | GO:0071277 | cellular response to calcium ion | 2月16日 | 82/18862 | 0.002153501 | 0.020718161 | 0.009751332 | FOS/FOSB | 2 |
| BP | GO:0007517 | muscle organ development | 3月16日 | 317/18862 | 0.002238474 | 0.021351601 | 0.010049471 | EGR1/FOS/ATF3 | 3 |
| BP | GO:0070371 | ERK1 and ERK2 cascade | 3月16日 | 320/18862 | 0.002299259 | 0.021677931 | 0.010203063 | DUSP1/ATF3/KLF4 | 3 |
| BP | GO:0042509 | regulation of tyrosine phosphorylation of STAT protein | 2月16日 | 85/18862 | 0.002311536 | 0.021677931 | 0.010203063 | SOCS3/IL6 | 2 |
| BP | GO:0071375 | cellular response to peptide hormone stimulus | 3月16日 | 325/18862 | 0.002402844 | 0.022346451 | 0.010517713 | NR4A1/SOCS3/NR4A2 | 3 |
| BP | GO:0007260 | tyrosine phosphorylation of STAT protein | 2月16日 | 88/18862 | 0.002474917 | 0.022826507 | 0.010743659 | SOCS3/IL6 | 2 |
| BP | GO:0060070 | canonical Wnt signaling pathway | 3月16日 | 333/18862 | 0.002574568 | 0.023470438 | 0.011046735 | EGR1/NR4A2/KLF4 | 3 |
| BP | GO:0070301 | cellular response to hydrogen peroxide | 2月16日 | 90/18862 | 0.002586796 | 0.023470438 | 0.011046735 | IL6/KLF4 | 2 |
| BP | GO:1904035 | regulation of epithelial cell apoptotic process | 2月16日 | 94/18862 | 0.002817619 | 0.02535857 | 0.011935415 | ZFP36/IL6 | 2 |
| BP | GO:0001666 | response to hypoxia | 3月16日 | 348/18862 | 0.002916773 | 0.025727152 | 0.012108893 | EGR1/PTGS2/NR4A2 | 3 |
| BP | GO:0048661 | positive regulation of smooth muscle cell proliferation | 2月16日 | 96/18862 | 0.002936549 | 0.025727152 | 0.012108893 | IL6/JUN | 2 |
| BP | GO:0014706 | striated muscle tissue development | 3月16日 | 351/18862 | 0.00298843 | 0.025727152 | 0.012108893 | EGR1/FOS/ATF3 | 3 |
| BP | GO:0032677 | regulation of interleukin-8 production | 2月16日 | 97/18862 | 0.00299689 | 0.025727152 | 0.012108893 | IL6/KLF4 | 2 |
| BP | GO:1990868 | response to chemokine | 2月16日 | 97/18862 | 0.00299689 | 0.025727152 | 0.012108893 | DUSP1/CXCL2 | 2 |
| BP | GO:1990869 | cellular response to chemokine | 2月16日 | 97/18862 | 0.00299689 | 0.025727152 | 0.012108893 | DUSP1/CXCL2 | 2 |
| BP | GO:0051052 | regulation of DNA metabolic process | 3月16日 | 353/18862 | 0.003036804 | 0.025870791 | 0.0121765 | DUSP1/IL6/KLF4 | 3 |
| BP | GO:1903426 | regulation of reactive oxygen species biosynthetic process | 2月16日 | 99/18862 | 0.003119322 | 0.02590551 | 0.01219284 | PTGS2/KLF4 | 2 |
| BP | GO:0010631 | epithelial cell migration | 3月16日 | 357/18862 | 0.003135007 | 0.02590551 | 0.01219284 | PTGS2/NR4A1/KLF4 | 3 |
| BP | GO:0007178 | transmembrane receptor protein serine/threonine kinase signaling pathway | 3月16日 | 358/18862 | 0.003159861 | 0.02590551 | 0.01219284 | EGR1/FOS/JUN | 3 |
| BP | GO:0042493 | response to drug | 3月16日 | 359/18862 | 0.003184838 | 0.02590551 | 0.01219284 | FOS/FOSB/JUN | 3 |
| BP | GO:0036293 | response to decreased oxygen levels | 3月16日 | 360/18862 | 0.003209937 | 0.02590551 | 0.01219284 | EGR1/PTGS2/NR4A2 | 3 |
| BP | GO:0090132 | epithelium migration | 3月16日 | 360/18862 | 0.003209937 | 0.02590551 | 0.01219284 | PTGS2/NR4A1/KLF4 | 3 |
| BP | GO:0046425 | regulation of receptor signaling pathway via JAK-STAT | 2月16日 | 101/18862 | 0.003244078 | 0.02590551 | 0.01219284 | SOCS3/IL6 | 2 |
| BP | GO:2000379 | positive regulation of reactive oxygen species metabolic process | 2月16日 | 101/18862 | 0.003244078 | 0.02590551 | 0.01219284 | PTGS2/KLF4 | 2 |
| BP | GO:0045639 | positive regulation of myeloid cell differentiation | 2月16日 | 102/18862 | 0.003307326 | 0.02590551 | 0.01219284 | FOS/JUN | 2 |
| BP | GO:0090130 | tissue migration | 3月16日 | 365/18862 | 0.003337274 | 0.02590551 | 0.01219284 | PTGS2/NR4A1/KLF4 | 3 |
| BP | GO:0032526 | response to retinoic acid | 2月16日 | 103/18862 | 0.003371152 | 0.02590551 | 0.01219284 | DUSP1/KLF4 | 2 |
| BP | GO:0032637 | interleukin-8 production | 2月16日 | 103/18862 | 0.003371152 | 0.02590551 | 0.01219284 | IL6/KLF4 | 2 |
| BP | GO:0032651 | regulation of interleukin-1 beta production | 2月16日 | 103/18862 | 0.003371152 | 0.02590551 | 0.01219284 | EGR1/IL6 | 2 |
| BP | GO:0070498 | interleukin-1-mediated signaling pathway | 2月16日 | 103/18862 | 0.003371152 | 0.02590551 | 0.01219284 | EGR1/IL6 | 2 |
| BP | GO:0001818 | negative regulation of cytokine production | 3月16日 | 367/18862 | 0.003389072 | 0.02590551 | 0.01219284 | ZFP36/IL6/KLF4 | 3 |
| BP | GO:0018108 | peptidyl-tyrosine phosphorylation | 3月16日 | 369/18862 | 0.003441366 | 0.026126285 | 0.012296752 | AREG/SOCS3/IL6 | 3 |
| BP | GO:0060537 | muscle tissue development | 3月16日 | 371/18862 | 0.003494156 | 0.026347826 | 0.012401024 | EGR1/FOS/ATF3 | 3 |
| BP | GO:0018212 | peptidyl-tyrosine modification | 3月16日 | 372/18862 | 0.003520738 | 0.026370095 | 0.012411505 | AREG/SOCS3/IL6 | 3 |
| BP | GO:0050678 | regulation of epithelial cell proliferation | 3月16日 | 374/18862 | 0.003574278 | 0.026592627 | 0.012516243 | AREG/ZFP36/NR4A1 | 3 |
| BP | GO:0002526 | acute inflammatory response | 2月16日 | 107/18862 | 0.003632225 | 0.026668175 | 0.012551801 | PTGS2/IL6 | 2 |
| BP | GO:2000278 | regulation of DNA biosynthetic process | 2月16日 | 107/18862 | 0.003632225 | 0.026668175 | 0.012551801 | DUSP1/KLF4 | 2 |
| BP | GO:0032611 | interleukin-1 beta production | 2月16日 | 108/18862 | 0.00369893 | 0.026980431 | 0.012698769 | EGR1/IL6 | 2 |
| BP | GO:1904892 | regulation of receptor signaling pathway via STAT | 2月16日 | 110/18862 | 0.00383406 | 0.027784484 | 0.01307721 | SOCS3/IL6 | 2 |
| BP | GO:0070482 | response to oxygen levels | 3月16日 | 385/18862 | 0.003877745 | 0.027919762 | 0.01314088 | EGR1/PTGS2/NR4A2 | 3 |
| BP | GO:0032102 | negative regulation of response to external stimulus | 3月16日 | 394/18862 | 0.004137491 | 0.029598973 | 0.013931228 | DUSP1/SOCS3/KLF4 | 3 |
| BP | GO:0001704 | formation of primary germ layer | 2月16日 | 115/18862 | 0.004181867 | 0.029725883 | 0.01399096 | DUSP1/KLF4 | 2 |
| BP | GO:1904019 | epithelial cell apoptotic process | 2月16日 | 116/18862 | 0.004253133 | 0.030041115 | 0.014139329 | ZFP36/IL6 | 2 |
| BP | GO:0002761 | regulation of myeloid leukocyte differentiation | 2月16日 | 118/18862 | 0.004397361 | 0.030606625 | 0.014405495 | FOS/JUN | 2 |
| BP | GO:0051101 | regulation of DNA binding | 2月16日 | 118/18862 | 0.004397361 | 0.030606625 | 0.014405495 | JUN/KLF4 | 2 |
| BP | GO:0002688 | regulation of leukocyte chemotaxis | 2月16日 | 119/18862 | 0.004470323 | 0.030606625 | 0.014405495 | DUSP1/IL6 | 2 |
| BP | GO:0032652 | regulation of interleukin-1 production | 2月16日 | 119/18862 | 0.004470323 | 0.030606625 | 0.014405495 | EGR1/IL6 | 2 |
| BP | GO:0120254 | olefinic compound metabolic process | 2月16日 | 119/18862 | 0.004470323 | 0.030606625 | 0.014405495 | EGR1/PTGS2 | 2 |
| BP | GO:0060964 | regulation of gene silencing by miRNA | 2月16日 | 121/18862 | 0.004617934 | 0.031424477 | 0.01479043 | ZFP36/IL6 | 2 |
| BP | GO:1903131 | mononuclear cell differentiation | 3月16日 | 411/18862 | 0.004656757 | 0.031496608 | 0.01482438 | EGR1/IL6/JUN | 3 |
| BP | GO:1903409 | reactive oxygen species biosynthetic process | 2月16日 | 123/18862 | 0.004767792 | 0.031972757 | 0.015048487 | PTGS2/KLF4 | 2 |
| BP | GO:1903706 | regulation of hemopoiesis | 3月16日 | 415/18862 | 0.004784454 | 0.031972757 | 0.015048487 | FOS/ZFP36/JUN | 3 |
| BP | GO:0060147 | regulation of posttranscriptional gene silencing | 2月16日 | 124/18862 | 0.004843561 | 0.032175082 | 0.015143715 | ZFP36/IL6 | 2 |
| BP | GO:0030099 | myeloid cell differentiation | 3月16日 | 419/18862 | 0.004914276 | 0.032297623 | 0.01520139 | FOS/ZFP36/JUN | 3 |
| BP | GO:0060966 | regulation of gene silencing by RNA | 2月16日 | 125/18862 | 0.004919889 | 0.032297623 | 0.01520139 | ZFP36/IL6 | 2 |
| BP | GO:0032612 | interleukin-1 production | 2月16日 | 126/18862 | 0.004996775 | 0.032610531 | 0.015348665 | EGR1/IL6 | 2 |
| BP | GO:0007612 | learning | 2月16日 | 130/18862 | 0.005309882 | 0.034452491 | 0.016215613 | FOS/JUN | 2 |
| BP | GO:0001819 | positive regulation of cytokine production | 3月16日 | 437/18862 | 0.005525061 | 0.035599945 | 0.016755681 | EGR1/PTGS2/IL6 | 3 |
| BP | GO:0050729 | positive regulation of inflammatory response | 2月16日 | 133/18862 | 0.005550529 | 0.035599945 | 0.016755681 | PTGS2/IL6 | 2 |
| BP | GO:0031667 | response to nutrient levels | 3月16日 | 451/18862 | 0.006030628 | 0.038458174 | 0.018100951 | ATF3/ZFP36/JUN | 3 |
| BP | GO:0044344 | cellular response to fibroblast growth factor stimulus | 2月16日 | 142/18862 | 0.006302133 | 0.03996125 | 0.018808398 | ZFP36/NR4A1 | 2 |
| BP | GO:0060968 | regulation of gene silencing | 2月16日 | 144/18862 | 0.006475153 | 0.040826391 | 0.01921559 | ZFP36/IL6 | 2 |
| BP | GO:0002262 | myeloid cell homeostasis | 2月16日 | 145/18862 | 0.006562476 | 0.041144515 | 0.019365321 | ZFP36/IL6 | 2 |
| BP | GO:0071774 | response to fibroblast growth factor | 2月16日 | 148/18862 | 0.006827686 | 0.042568143 | 0.020035374 | ZFP36/NR4A1 | 2 |
| BP | GO:0001667 | ameboidal-type cell migration | 3月16日 | 473/18862 | 0.006880126 | 0.042656782 | 0.020077093 | PTGS2/NR4A1/KLF4 | 3 |
| BP | GO:0043535 | regulation of blood vessel endothelial cell migration | 2月16日 | 150/18862 | 0.007007184 | 0.043204517 | 0.020334893 | PTGS2/KLF4 | 2 |
| BP | GO:1902107 | positive regulation of leukocyte differentiation | 2月16日 | 154/18862 | 0.00737261 | 0.044960833 | 0.021161531 | FOS/JUN | 2 |
| BP | GO:1903708 | positive regulation of hemopoiesis | 2月16日 | 154/18862 | 0.00737261 | 0.044960833 | 0.021161531 | FOS/JUN | 2 |
| BP | GO:0009267 | cellular response to starvation | 2月16日 | 157/18862 | 0.007652278 | 0.046412727 | 0.021844888 | ATF3/JUN | 2 |
| BP | GO:0032680 | regulation of tumor necrosis factor production | 2月16日 | 160/18862 | 0.007936719 | 0.046929553 | 0.022088141 | ZFP36/IL6 | 2 |
| BP | GO:0050728 | negative regulation of inflammatory response | 2月16日 | 160/18862 | 0.007936719 | 0.046929553 | 0.022088141 | SOCS3/KLF4 | 2 |
| BP | GO:0007626 | locomotory behavior | 2月16日 | 161/18862 | 0.008032589 | 0.046929553 | 0.022088141 | EGR1/NR4A2 | 2 |
| BP | GO:0032640 | tumor necrosis factor production | 2月16日 | 162/18862 | 0.008128987 | 0.046929553 | 0.022088141 | ZFP36/IL6 | 2 |
| BP | GO:1903555 | regulation of tumor necrosis factor superfamily cytokine production | 2月16日 | 164/18862 | 0.008323359 | 0.046929553 | 0.022088141 | ZFP36/IL6 | 2 |
| BP | GO:0001660 | fever generation | 1月16日 | 10/18862 | 0.008452366 | 0.046929553 | 0.022088141 | PTGS2 | 1 |
| BP | GO:0009629 | response to gravity | 1月16日 | 10/18862 | 0.008452366 | 0.046929553 | 0.022088141 | FOS | 1 |
| BP | GO:0019852 | L-ascorbic acid metabolic process | 1月16日 | 10/18862 | 0.008452366 | 0.046929553 | 0.022088141 | SLC2A3 | 1 |
| BP | GO:0031652 | positive regulation of heat generation | 1月16日 | 10/18862 | 0.008452366 | 0.046929553 | 0.022088141 | PTGS2 | 1 |
| BP | GO:0045647 | negative regulation of erythrocyte differentiation | 1月16日 | 10/18862 | 0.008452366 | 0.046929553 | 0.022088141 | ZFP36 | 1 |
| BP | GO:0070091 | glucagon secretion | 1月16日 | 10/18862 | 0.008452366 | 0.046929553 | 0.022088141 | IL6 | 1 |
| BP | GO:0070092 | regulation of glucagon secretion | 1月16日 | 10/18862 | 0.008452366 | 0.046929553 | 0.022088141 | IL6 | 1 |
| BP | GO:0072124 | regulation of glomerular mesangial cell proliferation | 1月16日 | 10/18862 | 0.008452366 | 0.046929553 | 0.022088141 | EGR1 | 1 |
| BP | GO:0072203 | cell proliferation involved in metanephros development | 1月16日 | 10/18862 | 0.008452366 | 0.046929553 | 0.022088141 | EGR1 | 1 |
| BP | GO:0097050 | type B pancreatic cell apoptotic process | 1月16日 | 10/18862 | 0.008452366 | 0.046929553 | 0.022088141 | IL6 | 1 |
| BP | GO:1902510 | regulation of apoptotic DNA fragmentation | 1月16日 | 10/18862 | 0.008452366 | 0.046929553 | 0.022088141 | IL6 | 1 |
| BP | GO:2000659 | regulation of interleukin-1-mediated signaling pathway | 1月16日 | 10/18862 | 0.008452366 | 0.046929553 | 0.022088141 | IL6 | 1 |
| BP | GO:0007259 | receptor signaling pathway via JAK-STAT | 2月16日 | 166/18862 | 0.008519829 | 0.047069948 | 0.02215422 | SOCS3/IL6 | 2 |
| BP | GO:0071706 | tumor necrosis factor superfamily cytokine production | 2月16日 | 167/18862 | 0.008618849 | 0.04738244 | 0.022301299 | ZFP36/IL6 | 2 |
| BP | GO:0038095 | Fc-epsilon receptor signaling pathway | 2月16日 | 169/18862 | 0.008818453 | 0.048242125 | 0.022705924 | FOS/JUN | 2 |
| BP | GO:0001659 | temperature homeostasis | 2月16日 | 171/18862 | 0.009020138 | 0.048467298 | 0.022811905 | EGR1/PTGS2 | 2 |
| BP | GO:0019371 | cyclooxygenase pathway | 1月16日 | 11/18862 | 0.009293908 | 0.048467298 | 0.022811905 | PTGS2 | 1 |
| BP | GO:0034115 | negative regulation of heterotypic cell-cell adhesion | 1月16日 | 11/18862 | 0.009293908 | 0.048467298 | 0.022811905 | KLF4 | 1 |
| BP | GO:0042416 | dopamine biosynthetic process | 1月16日 | 11/18862 | 0.009293908 | 0.048467298 | 0.022811905 | NR4A2 | 1 |
| BP | GO:0042541 | hemoglobin biosynthetic process | 1月16日 | 11/18862 | 0.009293908 | 0.048467298 | 0.022811905 | KLF4 | 1 |
| BP | GO:0045657 | positive regulation of monocyte differentiation | 1月16日 | 11/18862 | 0.009293908 | 0.048467298 | 0.022811905 | JUN | 1 |
| BP | GO:0070587 | regulation of cell-cell adhesion involved in gastrulation | 1月16日 | 11/18862 | 0.009293908 | 0.048467298 | 0.022811905 | KLF4 | 1 |
| BP | GO:0072110 | glomerular mesangial cell proliferation | 1月16日 | 11/18862 | 0.009293908 | 0.048467298 | 0.022811905 | EGR1 | 1 |
| BP | GO:1990440 | positive regulation of transcription from RNA polymerase II promoter in response to endoplasmic reticulum stress | 1月16日 | 11/18862 | 0.009293908 | 0.048467298 | 0.022811905 | ATF3 | 1 |
| BP | GO:2000551 | regulation of T-helper 2 cell cytokine production | 1月16日 | 11/18862 | 0.009293908 | 0.048467298 | 0.022811905 | IL6 | 1 |
| BP | GO:0097696 | receptor signaling pathway via STAT | 2月16日 | 175/18862 | 0.009429724 | 0.048751793 | 0.022945807 | SOCS3/IL6 | 2 |
| BP | GO:0001959 | regulation of cytokine-mediated signaling pathway | 2月16日 | 177/18862 | 0.009637611 | 0.048751793 | 0.022945807 | SOCS3/IL6 | 2 |
| BP | GO:0007565 | female pregnancy | 2月16日 | 177/18862 | 0.009637611 | 0.048751793 | 0.022945807 | FOS/FOSB | 2 |
| BP | GO:0007369 | gastrulation | 2月16日 | 179/18862 | 0.009847553 | 0.048751793 | 0.022945807 | DUSP1/KLF4 | 2 |
| BP | GO:0019083 | viral transcription | 2月16日 | 180/18862 | 0.009953292 | 0.048751793 | 0.022945807 | ZFP36/JUN | 2 |
| BP | GO:0071347 | cellular response to interleukin-1 | 2月16日 | 180/18862 | 0.009953292 | 0.048751793 | 0.022945807 | EGR1/IL6 | 2 |
| BP | GO:0008298 | intracellular mRNA localization | 1月16日 | 12/18862 | 0.010134781 | 0.048751793 | 0.022945807 | ZFP36 | 1 |
| BP | GO:0032352 | positive regulation of hormone metabolic process | 1月16日 | 12/18862 | 0.010134781 | 0.048751793 | 0.022945807 | EGR1 | 1 |
| BP | GO:0032494 | response to peptidoglycan | 1月16日 | 12/18862 | 0.010134781 | 0.048751793 | 0.022945807 | IL6 | 1 |
| BP | GO:0036462 | TRAIL-activated apoptotic signaling pathway | 1月16日 | 12/18862 | 0.010134781 | 0.048751793 | 0.022945807 | ATF3 | 1 |
| BP | GO:0044849 | estrous cycle | 1月16日 | 12/18862 | 0.010134781 | 0.048751793 | 0.022945807 | EGR1 | 1 |
| BP | GO:0060670 | branching involved in labyrinthine layer morphogenesis | 1月16日 | 12/18862 | 0.010134781 | 0.048751793 | 0.022945807 | SOCS3 | 1 |
| BP | GO:0060707 | trophoblast giant cell differentiation | 1月16日 | 12/18862 | 0.010134781 | 0.048751793 | 0.022945807 | SOCS3 | 1 |
| BP | GO:0061469 | regulation of type B pancreatic cell proliferation | 1月16日 | 12/18862 | 0.010134781 | 0.048751793 | 0.022945807 | NR4A1 | 1 |
| BP | GO:0070586 | cell-cell adhesion involved in gastrulation | 1月16日 | 12/18862 | 0.010134781 | 0.048751793 | 0.022945807 | KLF4 | 1 |
| BP | GO:0097201 | negative regulation of transcription from RNA polymerase II promoter in response to stress | 1月16日 | 12/18862 | 0.010134781 | 0.048751793 | 0.022945807 | JUN | 1 |
| BP | GO:1903624 | regulation of DNA catabolic process | 1月16日 | 12/18862 | 0.010134781 | 0.048751793 | 0.022945807 | IL6 | 1 |
| BP | GO:1903800 | positive regulation of production of miRNAs involved in gene silencing by miRNA | 1月16日 | 12/18862 | 0.010134781 | 0.048751793 | 0.022945807 | IL6 | 1 |
| BP | GO:0010565 | regulation of cellular ketone metabolic process | 2月16日 | 185/18862 | 0.010489636 | 0.049992178 | 0.023529614 | EGR1/PTGS2 | 2 |
| BP | GO:0006367 | transcription initiation from RNA polymerase II promoter | 2月16日 | 187/18862 | 0.010707728 | 0.049992178 | 0.023529614 | NR4A1/NR4A2 | 2 |
| BP | GO:0014745 | negative regulation of muscle adaptation | 1月16日 | 13/18862 | 0.010974985 | 0.049992178 | 0.023529614 | KLF4 | 1 |
| BP | GO:0031053 | primary miRNA processing | 1月16日 | 13/18862 | 0.010974985 | 0.049992178 | 0.023529614 | IL6 | 1 |
| BP | GO:0031392 | regulation of prostaglandin biosynthetic process | 1月16日 | 13/18862 | 0.010974985 | 0.049992178 | 0.023529614 | PTGS2 | 1 |
| BP | GO:0031650 | regulation of heat generation | 1月16日 | 13/18862 | 0.010974985 | 0.049992178 | 0.023529614 | PTGS2 | 1 |
| BP | GO:0034616 | response to laminar fluid shear stress | 1月16日 | 13/18862 | 0.010974985 | 0.049992178 | 0.023529614 | KLF4 | 1 |
| BP | GO:0035745 | T-helper 2 cell cytokine production | 1月16日 | 13/18862 | 0.010974985 | 0.049992178 | 0.023529614 | IL6 | 1 |
| BP | GO:0045741 | positive regulation of epidermal growth factor-activated receptor activity | 1月16日 | 13/18862 | 0.010974985 | 0.049992178 | 0.023529614 | AREG | 1 |
| BP | GO:0060213 | positive regulation of nuclear-transcribed mRNA poly(A) tail shortening | 1月16日 | 13/18862 | 0.010974985 | 0.049992178 | 0.023529614 | ZFP36 | 1 |
| BP | GO:1902043 | positive regulation of extrinsic apoptotic signaling pathway via death domain receptors | 1月16日 | 13/18862 | 0.010974985 | 0.049992178 | 0.023529614 | ATF3 | 1 |
| BP | GO:1902947 | regulation of tau-protein kinase activity | 1月16日 | 13/18862 | 0.010974985 | 0.049992178 | 0.023529614 | EGR1 | 1 |
| BP | GO:1904995 | negative regulation of leukocyte adhesion to vascular endothelial cell | 1月16日 | 13/18862 | 0.010974985 | 0.049992178 | 0.023529614 | KLF4 | 1 |
| CC | GO:0005667 | transcription regulator complex | 5月17日 | 409/19520 | 1.98E-05 | 0.000851809 | 0.000708972 | FOS/NR4A1/NR4A2/JUN/KLF4 | 5 |
| CC | GO:0000791 | euchromatin | 2月17日 | 36/19520 | 0.000441986 | 0.009502688 | 0.007909214 | JUN/KLF4 | 2 |
| MF | GO:0001228 | DNA-binding transcription activator activity, RNA polymerase II-specific | 8月17日 | 443/18337 | 2.19E-09 | 1.08E-07 | 4.20E-08 | EGR1/FOS/ATF3/NR4A1/FOSB/NR4A2/JUN/KLF4 | 8 |
| MF | GO:0001216 | DNA-binding transcription activator activity | 8月17日 | 447/18337 | 2.35E-09 | 1.08E-07 | 4.20E-08 | EGR1/FOS/ATF3/NR4A1/FOSB/NR4A2/JUN/KLF4 | 8 |
| MF | GO:0061629 | RNA polymerase II-specific DNA-binding transcription factor binding | 5月17日 | 271/18337 | 3.64E-06 | 0.000111494 | 4.34E-05 | FOS/NR4A1/NR4A2/JUN/KLF4 | 5 |
| MF | GO:0140297 | DNA-binding transcription factor binding | 5月17日 | 376/18337 | 1.78E-05 | 0.000410011 | 0.000159501 | FOS/NR4A1/NR4A2/JUN/KLF4 | 5 |
| MF | GO:0035259 | glucocorticoid receptor binding | 2月17日 | 12/18337 | 5.31E-05 | 0.000977077 | 0.000380099 | NR4A1/NR4A2 | 2 |
| MF | GO:0035014 | phosphatidylinositol 3-kinase regulator activity | 2月17日 | 20/18337 | 0.000152204 | 0.002333794 | 0.000907883 | SOCS3/KLF4 | 2 |
| MF | GO:0070412 | R-SMAD binding | 2月17日 | 22/18337 | 0.000184846 | 0.002429408 | 0.000945079 | FOS/JUN | 2 |
| MF | GO:0001102 | RNA polymerase II activating transcription factor binding | 2月17日 | 48/18337 | 0.000889924 | 0.009603484 | 0.003735909 | FOS/JUN | 2 |
| MF | GO:0004879 | nuclear receptor activity | 2月17日 | 52/18337 | 0.001043857 | 0.009603484 | 0.003735909 | NR4A1/NR4A2 | 2 |
| MF | GO:0098531 | ligand-activated transcription factor activity | 2月17日 | 52/18337 | 0.001043857 | 0.009603484 | 0.003735909 | NR4A1/NR4A2 | 2 |
| MF | GO:0005125 | cytokine activity | 3月17日 | 235/18337 | 0.001237323 | 0.009943915 | 0.003868342 | AREG/CXCL2/IL6 | 3 |
| MF | GO:1990841 | promoter-specific chromatin binding | 2月17日 | 58/18337 | 0.001297032 | 0.009943915 | 0.003868342 | EGR1/KLF4 | 2 |
| MF | GO:0035258 | steroid hormone receptor binding | 2月17日 | 77/18337 | 0.002272264 | 0.014295518 | 0.005561186 | NR4A1/NR4A2 | 2 |
| MF | GO:0046332 | SMAD binding | 2月17日 | 77/18337 | 0.002272264 | 0.014295518 | 0.005561186 | FOS/JUN | 2 |
| MF | GO:0033613 | activating transcription factor binding | 2月17日 | 78/18337 | 0.002330791 | 0.014295518 | 0.005561186 | FOS/JUN | 2 |
| MF | GO:0008013 | beta-catenin binding | 2月17日 | 85/18337 | 0.002760332 | 0.015871911 | 0.006174428 | NR4A2/KLF4 | 2 |
| MF | GO:0046982 | protein heterodimerization activity | 3月17日 | 324/18337 | 0.003092281 | 0.016734699 | 0.006510066 | ATF3/NR4A1/NR4A2 | 3 |
| MF | GO:0016922 | nuclear receptor binding | 2月17日 | 97/18337 | 0.003576597 | 0.018280385 | 0.007111363 | NR4A1/NR4A2 | 2 |
| MF | GO:0035257 | nuclear hormone receptor binding | 2月17日 | 140/18337 | 0.007301555 | 0.034051911 | 0.013246739 | NR4A1/NR4A2 | 2 |
| MF | GO:0070851 | growth factor receptor binding | 2月17日 | 141/18337 | 0.007402589 | 0.034051911 | 0.013246739 | AREG/IL6 | 2 |
| MF | GO:0005536 | glucose binding | 1月17日 | 10/18337 | 0.009234548 | 0.036300308 | 0.014121401 | SLC2A3 | 1 |
| MF | GO:0008330 | protein tyrosine/threonine phosphatase activity | 1月17日 | 10/18337 | 0.009234548 | 0.036300308 | 0.014121401 | DUSP1 | 1 |
| MF | GO:0048018 | receptor ligand activity | 3月17日 | 486/18337 | 0.009539776 | 0.036300308 | 0.014121401 | AREG/CXCL2/IL6 | 3 |
| MF | GO:0008083 | growth factor activity | 2月17日 | 162/18337 | 0.009669924 | 0.036300308 | 0.014121401 | AREG/IL6 | 2 |
| MF | GO:0030546 | signaling receptor activator activity | 3月17日 | 492/18337 | 0.009864214 | 0.036300308 | 0.014121401 | AREG/CXCL2/IL6 | 3 |
| MF | GO:0051427 | hormone receptor binding | 2月17日 | 173/18337 | 0.010966369 | 0.038804074 | 0.015095406 | NR4A1/NR4A2 | 2 |
| MF | GO:0017017 | MAP kinase tyrosine/serine/threonine phosphatase activity | 1月17日 | 13/18337 | 0.011989224 | 0.04085217 | 0.015892148 | DUSP1 | 1 |
| MF | GO:0071837 | HMG box domain binding | 1月17日 | 14/18337 | 0.012905846 | 0.042404922 | 0.016496194 | JUN | 1 |
| MF | GO:0033549 | MAP kinase phosphatase activity | 1月17日 | 15/18337 | 0.013821668 | 0.043734691 | 0.017013495 | DUSP1 | 1 |
| MF | GO:0035497 | cAMP response element binding | 1月17日 | 16/18337 | 0.014736689 | 0.043734691 | 0.017013495 | JUN | 1 |
| MF | GO:0046935 | 1-phosphatidylinositol-3-kinase regulator activity | 1月17日 | 16/18337 | 0.014736689 | 0.043734691 | 0.017013495 | SOCS3 | 1 |
| MF | GO:0046965 | retinoid X receptor binding | 1月17日 | 17/18337 | 0.015650912 | 0.044821146 | 0.017436144 | NR4A2 | 1 |
| MF | GO:0000979 | RNA polymerase II core promoter sequence-specific DNA binding | 1月17日 | 18/18337 | 0.016564337 | 0.044821146 | 0.017436144 | FOS | 1 |
| MF | GO:0045236 | CXCR chemokine receptor binding | 1月17日 | 18/18337 | 0.016564337 | 0.044821146 | 0.017436144 | CXCL2 | 1 |
| MF | GO:0019207 | kinase regulator activity | 2月17日 | 220/18337 | 0.0173094 | 0.045498995 | 0.017699838 | SOCS3/KLF4 | 2 |
| MF | GO:0005355 | glucose transmembrane transporter activity | 1月17日 | 21/18337 | 0.019299826 | 0.047988756 | 0.018668395 | SLC2A3 | 1 |
| MF | GO:0015149 | hexose transmembrane transporter activity | 1月17日 | 21/18337 | 0.019299826 | 0.047988756 | 0.018668395 | SLC2A3 | 1 |
