# Supplementary material for: The Discovery, Validation, and Function of Hypoxia-Related Gene Biomarkers for Obstructive Sleep Apnea
Source: Front Med (Lausanne). 2022 Mar 17;9:813459. doi: 10.3389/fmed.2022.813459 (PMC8970318; doi:10.3389/fmed.2022.813459)
Supplement: Supplementary Table 3 — KEGG pathway enrichment analysis of DEGs identified in GSE135917. [file Table_3.DOCX]

Supplementary Table 3. KEGG pathway enrichment analysis of DEGs identified in GSE135917

| ID | Description | GeneRatio | BgRatio | pvalue | p.adjust | qvalue | geneID | Count |
| --- | --- | --- | --- | --- | --- | --- | --- | --- |
| hsa04657 | IL-17 signaling pathway | 6月14日 | 94/8093 | 5.82E-09 | 6.58E-07 | 3.43E-07 | 5743/2353/2354/2920/3569/3725 | 6 |
| hsa04668 | TNF signaling pathway | 6月14日 | 112/8093 | 1.69E-08 | 9.52E-07 | 4.97E-07 | 5743/2353/2920/9021/3569/3725 | 6 |
| hsa05167 | Kaposi sarcoma-associated herpesvirus infection | 6月14日 | 194/8093 | 4.50E-07 | 1.69E-05 | 8.83E-06 | 5743/2353/7538/2920/3569/3725 | 6 |
| hsa05323 | Rheumatoid arthritis | 4月14日 | 93/8093 | 1.50E-05 | 0.000423196 | 0.000220764 | 2353/2920/3569/3725 | 4 |
| hsa05166 | Human T-cell leukemia virus 1 infection | 5月14日 | 222/8093 | 2.43E-05 | 0.000548841 | 0.000286307 | 1958/2353/7538/3569/3725 | 5 |
| hsa04380 | Osteoclast differentiation | 4月14日 | 128/8093 | 5.29E-05 | 0.000995524 | 0.000519323 | 2353/2354/9021/3725 | 4 |
| hsa04010 | MAPK signaling pathway | 5月14日 | 294/8093 | 9.34E-05 | 0.001508498 | 0.00078692 | 1843/374/2353/3164/3725 | 5 |
| hsa04932 | Non-alcoholic fatty liver disease | 4月14日 | 155/8093 | 0.000111562 | 0.001575819 | 0.000822039 | 2353/9021/3569/3725 | 4 |
| hsa05031 | Amphetamine addiction | 3月14日 | 69/8093 | 0.00020188 | 0.002534712 | 0.001322253 | 2353/2354/3725 | 3 |
| hsa05133 | Pertussis | 3月14日 | 76/8093 | 0.000268942 | 0.002871831 | 0.001498114 | 2353/3569/3725 | 3 |
| hsa05140 | Leishmaniasis | 3月14日 | 77/8093 | 0.000279559 | 0.002871831 | 0.001498114 | 5743/2353/3725 | 3 |
| hsa05210 | Colorectal cancer | 3月14日 | 86/8093 | 0.000387532 | 0.003417953 | 0.001783003 | 374/2353/3725 | 3 |
| hsa05417 | Lipid and atherosclerosis | 4月14日 | 215/8093 | 0.000393216 | 0.003417953 | 0.001783003 | 2353/2920/3569/3725 | 4 |
| hsa04933 | AGE-RAGE signaling pathway in diabetic complications | 3月14日 | 100/8093 | 0.000603603 | 0.004493228 | 0.002343929 | 1958/3569/3725 | 3 |
| hsa05142 | Chagas disease | 3月14日 | 102/8093 | 0.000639622 | 0.004493228 | 0.002343929 | 2353/3569/3725 | 3 |
| hsa04620 | Toll-like receptor signaling pathway | 3月14日 | 104/8093 | 0.000676992 | 0.004493228 | 0.002343929 | 2353/3569/3725 | 3 |
| hsa04625 | C-type lectin receptor signaling pathway | 3月14日 | 104/8093 | 0.000676992 | 0.004493228 | 0.002343929 | 5743/3569/3725 | 3 |
| hsa04928 | Parathyroid hormone synthesis, secretion and action | 3月14日 | 106/8093 | 0.000715735 | 0.004493228 | 0.002343929 | 1958/2353/4929 | 3 |
| hsa04659 | Th17 cell differentiation | 3月14日 | 108/8093 | 0.000755872 | 0.004495452 | 0.002345089 | 2353/3569/3725 | 3 |
| hsa05135 | Yersinia infection | 3月14日 | 137/8093 | 0.001506724 | 0.008069024 | 0.004209272 | 2353/3569/3725 | 3 |
| hsa05162 | Measles | 3月14日 | 139/8093 | 0.001570961 | 0.008069024 | 0.004209272 | 2353/3569/3725 | 3 |
| hsa05418 | Fluid shear stress and atherosclerosis | 3月14日 | 139/8093 | 0.001570961 | 0.008069024 | 0.004209272 | 1843/2353/3725 | 3 |
| hsa04921 | Oxytocin signaling pathway | 3月14日 | 154/8093 | 0.002108301 | 0.010358175 | 0.005403426 | 5743/2353/3725 | 3 |
| hsa05161 | Hepatitis B | 3月14日 | 162/8093 | 0.002436561 | 0.011472141 | 0.005984535 | 2353/3569/3725 | 3 |
| hsa05030 | Cocaine addiction | 2月14日 | 49/8093 | 0.003119866 | 0.014101793 | 0.007356315 | 2354/3725 | 2 |
| hsa04621 | NOD-like receptor signaling pathway | 3月14日 | 184/8093 | 0.003498336 | 0.015204307 | 0.00793145 | 2920/3569/3725 | 3 |
| hsa05134 | Legionellosis | 2月14日 | 57/8093 | 0.004200782 | 0.017116223 | 0.008928817 | 2920/3569 | 2 |
| hsa05130 | Pathogenic Escherichia coli infection | 3月14日 | 197/8093 | 0.004241188 | 0.017116223 | 0.008928817 | 2353/3569/3725 | 3 |
| hsa05207 | Chemical carcinogenesis - receptor activation | 3月14日 | 212/8093 | 0.005210561 | 0.020303219 | 0.010591339 | 2353/3725/9314 | 3 |
| hsa05321 | Inflammatory bowel disease | 2月14日 | 65/8093 | 0.005431631 | 0.020459142 | 0.010672678 | 3569/3725 | 2 |
| hsa04917 | Prolactin signaling pathway | 2月14日 | 70/8093 | 0.006275387 | 0.022159961 | 0.011559924 | 2353/9021 | 2 |
| hsa05120 | Epithelial cell signaling in Helicobacter pylori infection | 2月14日 | 70/8093 | 0.006275387 | 0.022159961 | 0.011559924 | 2920/3725 | 2 |
| hsa05171 | Coronavirus disease - COVID-19 | 3月14日 | 232/8093 | 0.006698217 | 0.022936317 | 0.011964916 | 2353/3569/3725 | 3 |
| hsa05132 | Salmonella infection | 3月14日 | 249/8093 | 0.008145128 | 0.027070573 | 0.014121584 | 2353/3569/3725 | 3 |
| hsa04662 | B cell receptor signaling pathway | 2月14日 | 82/8093 | 0.008528044 | 0.027533398 | 0.014363021 | 2353/3725 | 2 |
| hsa04012 | ErbB signaling pathway | 2月14日 | 85/8093 | 0.009140362 | 0.028690581 | 0.014966675 | 374/3725 | 2 |
| hsa05235 | PD-L1 expression and PD-1 checkpoint pathway in cancer | 2月14日 | 89/8093 | 0.009986752 | 0.03050008 | 0.015910615 | 2353/3725 | 2 |
| hsa04658 | Th1 and Th2 cell differentiation | 2月14日 | 92/8093 | 0.010643777 | 0.031486388 | 0.01642513 | 2353/3725 | 2 |
| hsa04912 | GnRH signaling pathway | 2月14日 | 93/8093 | 0.010866983 | 0.031486388 | 0.01642513 | 1958/3725 | 2 |
| hsa01522 | Endocrine resistance | 2月14日 | 98/8093 | 0.012014196 | 0.032323908 | 0.016862029 | 2353/3725 | 2 |
| hsa04925 | Aldosterone synthesis and secretion | 2月14日 | 98/8093 | 0.012014196 | 0.032323908 | 0.016862029 | 3164/4929 | 2 |
| hsa05231 | Choline metabolism in cancer | 2月14日 | 98/8093 | 0.012014196 | 0.032323908 | 0.016862029 | 2353/3725 | 2 |
| hsa04061 | Viral protein interaction with cytokine and cytokine receptor | 2月14日 | 100/8093 | 0.012487505 | 0.032816001 | 0.017118734 | 2920/3569 | 2 |
| hsa05146 | Amoebiasis | 2月14日 | 102/8093 | 0.012968975 | 0.033061229 | 0.017246659 | 2920/3569 | 2 |
| hsa04064 | NF-kappa B signaling pathway | 2月14日 | 104/8093 | 0.013458553 | 0.033061229 | 0.017246659 | 5743/2920 | 2 |
| hsa04660 | T cell receptor signaling pathway | 2月14日 | 104/8093 | 0.013458553 | 0.033061229 | 0.017246659 | 2353/3725 | 2 |
| hsa04931 | Insulin resistance | 2月14日 | 108/8093 | 0.01446182 | 0.034769908 | 0.018138005 | 9021/3569 | 2 |
| hsa04726 | Serotonergic synapse | 2月14日 | 115/8093 | 0.016293787 | 0.038358291 | 0.020009914 | 1843/5743 | 2 |
| hsa04935 | Growth hormone synthesis, secretion and action | 2月14日 | 119/8093 | 0.017383477 | 0.040088426 | 0.020912453 | 2353/9021 | 2 |
| hsa04926 | Relaxin signaling pathway | 2月14日 | 129/8093 | 0.020240876 | 0.04574438 | 0.023862927 | 2353/3725 | 2 |
| hsa04151 | PI3K-Akt signaling pathway | 3月14日 | 354/8093 | 0.021085383 | 0.046718594 | 0.024371134 | 374/3164/3569 | 3 |
| hsa04210 | Apoptosis | 2月14日 | 136/8093 | 0.022351602 | 0.048571751 | 0.025337849 | 2353/3725 | 2 |
| hsa04915 | Estrogen signaling pathway | 2月14日 | 138/8093 | 0.02297103 | 0.048975969 | 0.025548712 | 2353/3725 | 2 |
